# Primary cell wall inspired micro containers as a step towards a synthetic plant cell

**DOI:** 10.1038/s41467-020-14718-x

**Published:** 2020-02-19

**Authors:** T. Paulraj, S. Wennmalm, D. C. F. Wieland, A. V. Riazanova, A. Dėdinaitė, T. Günther Pomorski, M. Cárdenas, A. J. Svagan

**Affiliations:** 1 KTH Royal Institute of Technology, Department of Fibre and Polymer Technology, Teknikringen 56, 100 44 Stockholm, Sweden; 2 KTH Royal Institute of Technology, SciLifeLab, Department of Applied Physics, Biophysics, Tomtebodavägen 23a, 171 65 Solna, Sweden; 3 Helmholtz-Zentrum Geesthacht: Centre for Materials and Costal Research, Institute of Materials Research, Max-Planck-Straße 1, 21502 Geesthacht, Germany; 4 KTH Royal Institute of Technology, Deptartment of Chemistry, Division of Surface and Corrosion Science, Drottning Kristinas väg 51, 100 44 Stockholm, Sweden; 5 RISE Research Institutes of Sweden, Division of Bioscience and Materials, 114 86 Stockholm, Sweden; 6 Ruhr University Bochum, Faculty of Chemistry and Biochemistry, Department of Molecular Biochemistry, Universitätsstraße 150, 44780 Bochum, Germany; 7 University of Copenhagen, Department for Plant and Environmental Sciences, Thorvaldsensvej 40, 1871 Frederiksberg C, Denmark; 8 Malmö University, Biofilm – Research Center for Biointerfaces and Department of Biomedical Science, 20506 Malmö, Sweden

**Keywords:** Membrane structure and assembly, Cell wall, Biomaterials - cells

## Abstract

The structural integrity of living plant cells heavily relies on the plant cell wall containing a nanofibrous cellulose skeleton. Hence, if synthetic plant cells consist of such a cell wall, they would allow for manipulation into more complex synthetic plant structures. Herein, we have overcome the fundamental difficulties associated with assembling lipid vesicles with cellulosic nanofibers (CNFs). We prepare plantosomes with an outer shell of CNF and pectin, and beneath this, a thin layer of lipids (oleic acid and phospholipids) that surrounds a water core. By exploiting the phase behavior of the lipids, regulated by pH and Mg^2+^ ions, we form vesicle-crowded interiors that change the outer dimension of the plantosomes, mimicking the expansion in real plant cells during, e.g., growth. The internal pressure enables growth of lipid tubules through the plantosome cell wall, which paves the way to the development of hierarchical plant structures and advanced synthetic plant cell mimics.

## Abstract (web summary)

Assembling synthetic plant cell is difficult due to the presence of primary cell wall. Here, the authors describe the assembly of lipid-containing bodies that can be coated with cellulose and pectin, and show how these so-called plantosomes can be manipulated by changing surrounding milieu.

## Introduction

The field of synthetic biology has widened our understanding of modern animal cells, and provided inspiration and innovative ideas for material chemistry^1–4^. Several attempts have been made to construct synthetic animal cells^5–7^. However, examples of synthetic plant cells, with both cell wall and plasma membrane mimics, have to our knowledge not been reported. This is surprising, since plant cells have well-defined structures (plasmodesmata) that serve as communication bridges across individual cells^8–10^, and a synthetic plant cell could therefore serve as a simple model to understand intracellular communication. A major challenge when constructing synthetic plant cells is to prepare a continuous cellulose microfibril layer on top of biomimetic plasma membranes^11^. In natural plant cells, a cell wall surrounds the plasma membrane^12^. In parenchyma cells, the wall is a thin primary cell wall that encompasses a nanofibrous cellulosic network, pectin, hemicellulose, and minor fractions of structural proteins^13^. The so-called cellulosic microfibrils, typically having a width of ~4 nm and several micrometers in length, are essential for the mechanical and structural integrity of the plant cell wall^13^. Cellulosic microfibrils can be extracted from plants in the form of cellulosic nanofibers (CNFs)^14^. However, due to their considerable length and semicrystalline nature, it has not been possible to assemble CNFs on top of vesicles; lipid vesicles typically have diameters below the micrometer range, while CNFs consist of stiff crystalline segments (~300 nm in wood) and only allow coating formation on top of spherical structures above ~600 nm in diameter^15^. Natural plant cells are 10–100 µm in size^12^, and thus to mimic plant cells, micrometer-sized vesicles are necessary. Even though reconstitution of polymer and polysaccharides on giant unilamellar phospholipid vesicles (GUVs) has been demonstrated in the past, to the authors’ knowledge, there are no studies to date that report GUVs coated with a continuous and dense layer of CNFs. This is because GUVs are quite fragile. CNFs, on the other hand, which form a viscous suspension in water (even at low concentrations), are highly entangled and difficult to process. In other words, CNF suspensions present other challenges compared to dissolved polymer solutions. Recently, more robust GUVs were successfully prepared, by using an outer stabilizing layer of block copolymers to first encapsulate several small vesicles in water-in-oil droplets, followed by the fusion of vesicles into one single GUV inside each water droplet^16^. Unfortunately, such a protocol cannot be used with natural CNFs (where CNFs take the role of the block copolymer), as CNFs can only be dispersed in water. Another strategy is to exploit the unique colloidal and physicochemical properties of nanocellulose at oil/water interfaces, which can be used to self-assemble a dense CNF layer at such interfaces^15,17^. In this study, we use precisely these properties to obtain a continuous layer of CNFs via a modified production protocol.

Herein, the primary plant cell wall polysaccharides nanocellulose and pectin are combined with oleic acid (OA), oleate, and structural plant phospholipids to generate plant-cell-inspired microcapsules, which we call plantosomes. OA has previously been used in the assembly of models of primitive cells, so-called protocells^18,19^. OA and oleate show rich phase behavior in aqueous media^20–22^, which can be utilized together with phospholipids, for an alternative fabrication route of plant cell mimics. Similar to the turgor pressure mechanism in real plant cells, the phase behavior of the OA/oleate-rich interior of plantosomes can be utilized to expand the microcapsules. Moreover, by tuning the formation conditions, the plantosome interior can be filled with a crowded lipid-based milieu that also extends through the polysaccharide capsule wall in the form of lipid tubular structures. The present study represents an important step toward fabrication of advanced synthetic plant cells, and studies of such synthetic cells in physiologically relevant settings might improve our understanding of the evolution of plant cells.

## Results

### Formation of CNF/pectin microcapsules with OA/oleate cores

**Fig. 1:**
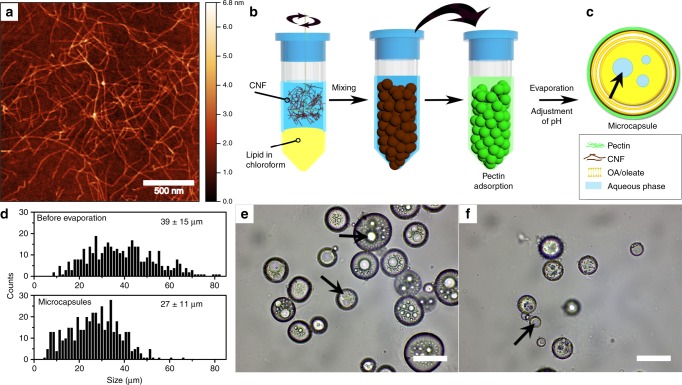
Polysaccharide assembly on the surface of lipid droplets. **a** Representative AFM image of the cationic CNFs (derived from three experiments). **b** Schematic representation of the preparation of CNF/pectin-stabilized microcapsules with OA/oleate cores. **c** Proposed organization within the obtained microcapsule, including the organization of the OA/oleate beneath the outer CNF/pectin shell. In **b** and **c**: water—blue, lipid—yellow, pectin—green, and CNF—brown. The size distribution **d** and corresponding representative bright field images of CNF/pectin-stabilized oil droplets prior to chloroform evaporation **e** and for microcapsules **f**. Data in **d** were collected from four experiments, and histograms includes *n* = 402 CNF/pectin-stabilized oil droplets before chloroform evaporation and *n* = 447 microcapsules. The average diameters and s.d. are reported. Arrows in **c**, **e**, and **f** point to encapsulated water droplets. Height bar: 0 − 6.8 nm **a**. Scale bars: 500 nm **a** and 50 µm **e**, **f**.

First, we studied the self-assembly of OA/oleate and the polysaccharides to understand how the phase behavior of OA could be exploited to make artificial plant cells. OA forms an oil-in-water emulsion at low pH (<7), but cubic, lamellar, and micellar phases upon increasing the pH to, respectively, 7.5, 8–9, or even higher^20^. In the present study, cationic CNFs, extracted from wood pulp, were used in the fabrication of plant cell mimics (Fig. 1a). A 288 mM OA solution in chloroform was emulsified in the presence of an aqueous CNF suspension (0.059 wt%), using a 1:1 volume ratio (Fig. 1b). The nanofibers accumulated at the oil/water interface and stabilized the emulsion (Supplementary Figs. 1–3)^15^. In a further step, sugar beet pectin was adsorbed on top of the CNF layer (Supplementary Figs. 4 and 5, Supplementary Note 1), further providing stability and allowing microcapsule with OA/oleate cores to evolve. Both CNF and pectin were pivotal for stability during the microcapsule formation (Supplementary Figs. 1–4, Supplementary Method 1, Supplementary Note 1). The final microcapsules were formed by evaporating the chloroform, followed by adjusting the pH of the microcapsule suspension to 2 and then 6.5 (details in Supplementary Fig. 3, Supplementary Method 2, Supplementary Note 2). A schematic representation of the final microcapsule is shown in Fig. 1c. Prior to evaporation of chloroform, the CNF/pectin-stabilized oil droplets contained both chloroform and OA, and had a diameter of 39 ± 15 µm (Fig. 1d). In addition, the interior of the oil-phase occasionally contained water droplets (arrow in Fig. 1e). The final microcapsules, on the other hand, were much smaller, 27 ± 11 µm (Fig. 1d, f), which corresponds to a significant volume decrease of 67 vol% and shrinking of the outer CNF/pectin wall area with 52%, on average. The large volume shrinkage, and the pH (6.5) of the suspension, suggests a microcapsule interior presenting high OA content in its protonated form^20^ and some occasional water droplets.

**Fig. 2:**
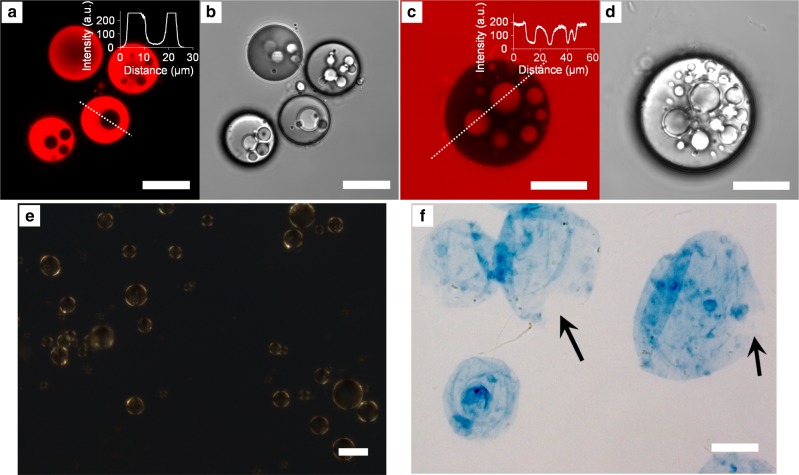
Microscopy images of microcapsules with predominately lipid in the interior. CLSM images of **a**, **b** microcapsules exposed to rhodamine 6 G (Rh-6G, 0.01 mg mL^−1^) and **c**, **d** sulforhodamine 101 (SR-101, 0.5 mg mL^−1^). Insets show intensity line profiles obtained from the marked lines. **a**, **c** and **b**, **d** are fluorescence and transmission images, respectively. **e** The OA/oleate lipids were organized concentrically in the periphery (below the CNF/pectin capsule wall) of the microcapsules, observed as Maltese crosses in POM. In **f**, light microscopy image of empty and collapsed microcapsule walls (i.e., devoid of OA/oleate cores). The capsules were emptied by increasing the pH, see Supplementary Movie 1. The capsule walls were stained blue with calcofluor-white stain. The arrows point to burst cavities. All images are representative of three experiments. Scale bars: 20 µm **a**–**d** and **f**, and 50 µm **e**.

To identify the interior oil and water parts, the microcapsules were exposed to dyes that labeled the hydrophobic lipid core regions (rhodamine 6 G, Rh-6G)^23^ and the aqueous regions (sulforhodamine 101, SR-101)^23^ of the microcapsule interior. These dyes confirmed that the microcapsule interior consisted mainly of lipid (Fig. 2a, b, stained with Rh-6G), with a minor fraction of water droplets (Fig. 2c, d, SR-101). Polarized optical microscopy (POM, Fig. 2e) revealed Maltese crosses at the outer rim of the microcapsules, indicative for concentrically organized (lamellar) lipids.

To reveal the presence of the encasing CNF/pectin wall, the lipids were removed from the microcapsule interior (Supplementary Movie 1) and the remaining microcapsule walls were stained with a beta-glucan-binding dye (calcofluor-white stain; Fig. 2f). The lipid core removal was achieved by increasing the pH, which led to the lipid solubilization into vesicles and then micelles, which could escape through the microcapsule wall. In the process, the interior volume expanded with a concomitant microcapsule radius increase, signifying a large extensibility of the encasing CNF/pectin wall. The radius and volume of the microcapsules in Supplementary Movie 1 increased with ca. 41% and 180 vol% on average, respectively, during the expansion, which is in the same order as the observed shrinkage during microcapsule preparation (Fig. 1d). The increase in the outer capsule wall area was 98% on average. Occasionally microcapsules also burst during the rapid expansion (the arrows in Fig. 2f point to such burst cavities).

### Formation of plantosomes

**Fig. 3:**
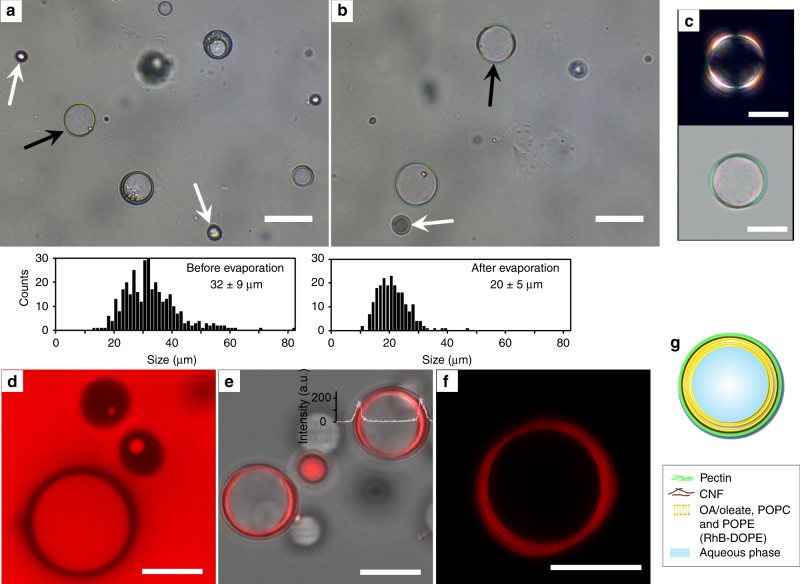
Plantosomes–microcapsules with thin interior lipid layers and large water-filled cavities. **a** Bright field image prior to chloroform evaporation. A mix of CNF/pectin-stabilized chloroform/lipid droplets devoid of water droplets or with varying sizes of water droplets, some of which were very large and filled out a large volume of the inner core (plantosomes). The lipid phase consisted of OA, POPE, and POPC. **b** After chloroform evaporation, the final population consisted of capsules with a similar composition as in **a**. Black and white arrows point to plantosomes and microcapsules devoid of water in the interior, respectively. Histograms: the size distribution of plantosomes prior (*n* = 356) and after chloroform evaporation (*n* = 218). Data derived from four experiments and images in **a** and **b** are representative images in these experiments. The average diameters and s.d. are reported **c** POM (bright field) image showing the organization of lipids in a plantosome. **d** CLSM images of plantosome and microcapsules showing the interior water parts stained with SR-101 (0.5 mg mL^−1^). **e** Combined fluorescence–transmission image of plantosomes and microcapsules containing Rh-DOPE (red) in the lipid phase. A superimposed fluorescence intensity profile is included for one plantosome. **f** CLSM image of a single plantosome containing Rh-DOPE (red) in the lipid phase. The lipids are organized in a couple of concentric rings in the periphery. Images in **c**–**f** are representative of three experiments. **g** Schematic representation of the cross-section of a plantosome. Scale bars: 20 µm **a**, **b**, and 10 µm **c**–**f**.

The microcapsules in Figs. 1 and 2 contained mostly lipid in their cores. Living parenchyma cells, on the other hand, contain a water-based cytoplasm enclosed by the plasma membrane, and ~1 wt% of lipid (hydrated state)^24^. To include a higher fraction of water and less lipids in the interior of the microcapsules, a small amount of phospholipids (0.22 mol% with respect to the total lipid amount) was also dissolved in the chloroform solution that was used in the production protocol. In this way, a population of microcapsules, consisting of CNF/pectin shells with very thin lipid layers beneath the shell and large water droplets in the interior, was attained (Fig. 3). We call them plantosomes. A mixture (1:5 mol ratio) of 1-palmitoyl-2-oleoyl-*sn*-glycero-3-phosphoethanolamine (POPE) and 1-palmitoyl-2-oleoyl-*sn*-glycero-3-phosphocholine (POPC) was used in the self-assembly process. These phospholipids are naturally found in the plasma membrane of plant cells and plasmodesmata^9^, representing 68–80% of the structural phospholipids^25^. Only a small amount of phospholipid was necessary (0.3 mM of phospholipid in the chloroform phase) to achieve the spontaneous self-assembly into plantosomes. The final microcapsule suspension was, still, a mixture of capsules with varying sizes of water-filled cavities (plantosomes, black arrows), and in some cases, the water cavity was missing (microcapsules, white arrows Fig. 3a, b). The diameter of the plantosomes prior to and after chloroform evaporation was 32 ± 9 µm and 20 ± 5 µm, respectively (histograms in Fig. 3). The decrease in the average diameter was both due to chloroform evaporation and that larger plantosomes burst during the evaporation.

After chloroform evaporation, the pH of the capsule suspension was around 5.8–5.9, which means that the OA was mainly present in its protonated form within the lipid layer^20–22^. The lipids were found in one or a couple of concentric rings in the periphery of the plantosomes (Fig. 3c, f). The presence of the large water compartments in the interior in the plantosomes was further verified by exposing them to the permeable water-soluble dye SR-101 (Fig. 3d). In Fig. 3e, f, the plantosomes and microcapsule also contained a rhodamine-labeled phospholipid (1,2-dioleoyl-*sn*-glycero-3-phosphoethanolamine-*N*-(lissamine rhodamine B sulfonyl) Rh-DOPE. A schematic representation of a plantosome is shown in Fig. 3g.

**Fig. 4:**
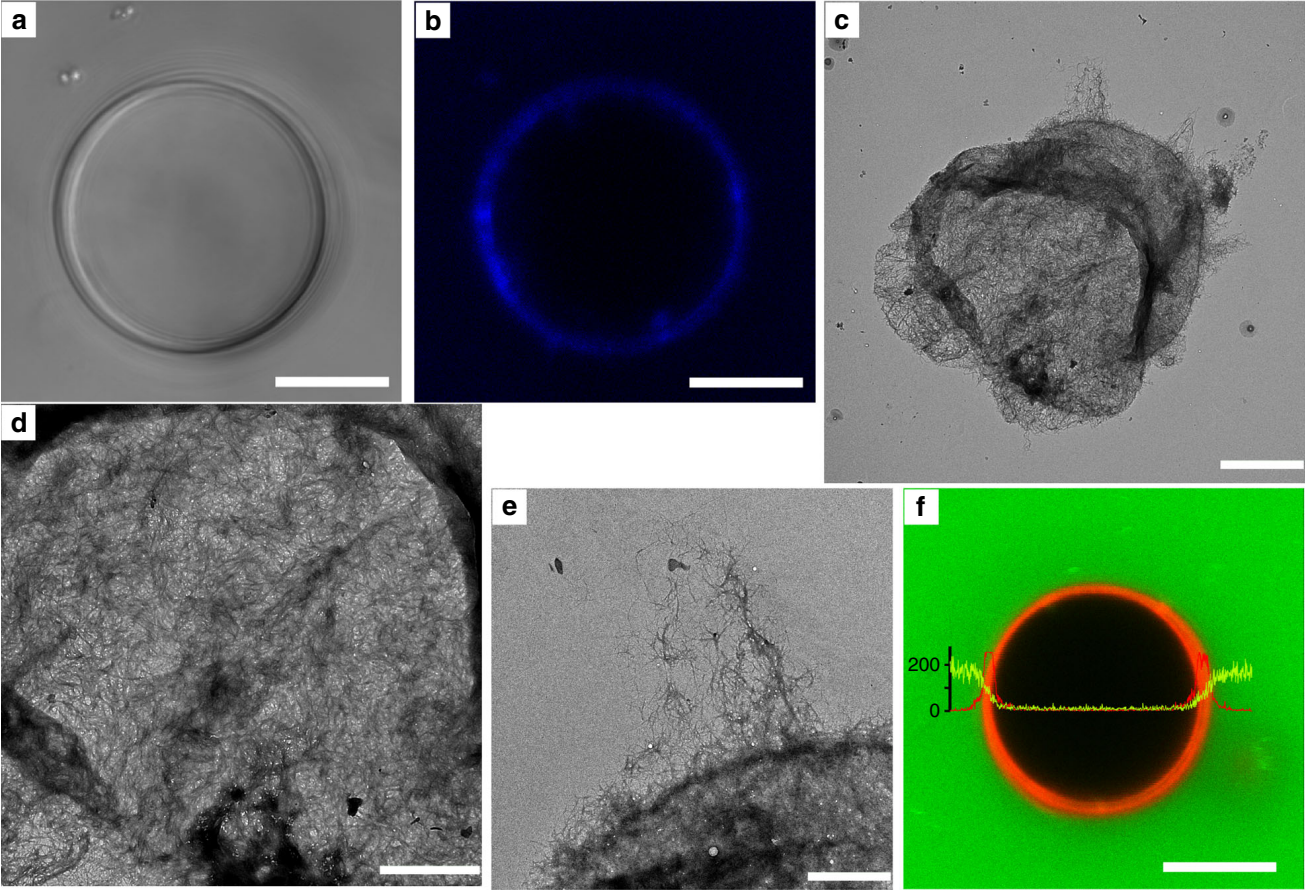
The nanostructure of the encasing CNF/pectin wall. CLSM transmission **a** and fluorescence **b** images of a plantosome stained with calcofluor-white (blue). TEM micrographs **c**–**e** of the remaining (dry) pectin/CNF shell after removal of lipids (OA, POPC, and POPE) from the interior of plantosomes/microcapsules. A burst cavity was created during the lipid removal. Images in **d** and **e** are high-resolution images of the shell in **c**. In **f**, fluorescence intensity profile for a plantosome exposed to FITC–dextran ($\bar M_w$= 4 kDa, 1 mg mL^−1^, green). The lipid phase contained Rh-DOPE (red). Images **a**–**b** and **c**–**f** are representative of two and three repeated experiments, respectively. Scale bars: 10 µm **a**, **b**, 5 µm **c**, 2 µm **d**, 1 µm **e**, and 10 µm **f**.

Confocal laser scanning microscopy (CLSM) studies of calcofluor-white stained plantosomes (Fig. 4a, b) provided information about the organization of the CNFs in the outer encasing shell. To elucidate details of the nanoscale structure of the CNF/pectin shell, we performed transmission electron microscope (TEM) and scanning electron microscope (SEM) studies of the remaining shell after lipid removal (Fig. 4c–e, Supplementary Fig. 6, Supplementary Note 3). During lipid removal, the plantosomes and microcapsules expanded, and the shells were stretched, as described previously. A shell that burst during lipid release was selected for TEM imaging (Fig. 4c–e), a technique that allowed better visualization of CNFs. High-magnification images of different parts of the remaining shell in Fig. 4c are given in Fig. 4d, e. These images revealed a dense structure consisting of a network of slender nanofibrous cellulose in a pectin matrix, which demonstrated successful self-assembly of CNFs and pectin into a dense shell. This shell represents a simple model of the primary cell wall in real plant cells, which consists of a network of hemicellulose-crosslinked cellulose microfibrils embedded in a pectin matrix^13,26^. Nano-sized pores, on average 18 ± 12 nm (*n* = 120) in the dry state, were also observed in the present shell structures (best observed in the SEM images in Supplementary Fig. 6a, b) after lipid removal. Occasionally larger pores, that were several of tens of nanometers in diameter, also could be observed (Supplementary Fig. 6b). These pores are larger than for the primary cell wall in living plant cells, which consists of pores ranging from 3.5 to 5.2 nm (in the wet state)^27^. However, it is unclear if the pores were formed during self-assembly or were a consequence of lipid removal.

In Fig. 4f, a plantosome is presented prior to expansion, where the lipid layers are still intact. We observed that plantosomes were partially permeable to small fluorescein isothiocyanate (FITC)–dextran molecules ($\bar M_w$= 4 kDa): a fraction of FITC–dextran could enter the space between the concentric lipid rings but no FITC-labeled dextran was observed in the interior water core of plantosomes. Thus, FITC-labeled dextrans with a hydrodynamic diameter of ~3 nm, could permeate though the encasing CNF/pectin shell but not the lipid barrier.

### Expansion and formation of lipid tubular structures

Plant cells are normally under turgor pressure, which tightly presses the plasma membrane against the cell wall^12^. The cell wall, on the other hand, preserves the plant cell and protects it from bursting. At the same time, plant cells are able to allow tubular membrane structures to cross their cell wall and act as a communication bridge across cells. The turgor pressure is controlled by the vacuoles, which are fluid-filled compartments that are able to expand the plant cells via osmotic uptake of water. Such cell enlargement is critical during, e.g., cell growth.

**Fig. 5:**
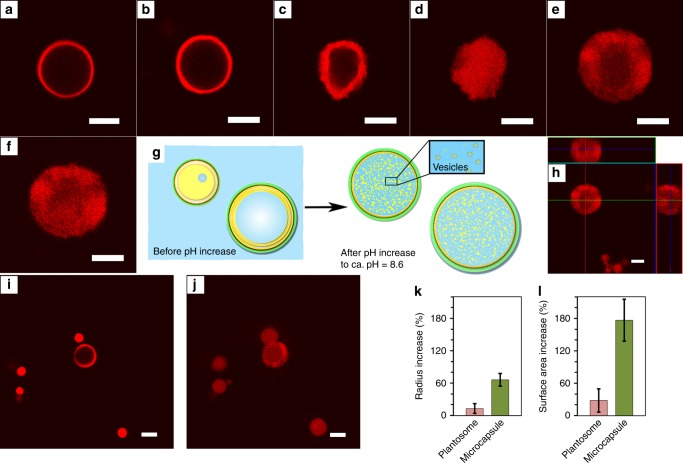
Formation of vesicles inside plantosomes. CLSM images of plantosome in **a** 100 mM NaCl solution, **b** transferred into 0.19 M ammonium acetate solution (with 100 mM NaCl) at pH 6.5. Increasing the pH in a step-wise manner **c** to 8.0 **d**, 8.3, and finally **e** to 8.6. After the final pH increase, an expanded plantosome is created. In **f**, after 1 h at pH 8.6. **g** Proposed mechanism for the formation of vesicles in the interior. **h** Orthogonal view of the expanded plantosome in **e**. In **c**–**h**, the medium contained 0.2 M ammonium acetate, 100 mM NaCl. The lipid phase contained OA/oleate, POPE, POPC, and Rh-DOPE (red). The images for the experiment in **a**–**f** are representative of seven repeated experiments. In **i** and **j**, the same type of experiment repeated for a plantosome and microcapsules (without water cavities). The pH is 6.5 **i** and 8.6 **j**. Images in **i**–**j** are representative of five experiments. The average increase in radius **k** and surface area **l** for a population of microcapsules (*n* = 22 microcapsules, obtained from five experiments) and plantosomes (*n* = 9 plantosomes, obtained from nine experiments). Data are presented as mean ± s.d. Scale bars: 10 µm. Source data underlying **k**, **l** are provided as a Source Data file.

We therefore tested whether it is possible to expand also our plantosomes. As our plantosomes have no vacuoles, we aimed to create a crowded lipid milieu in the interior. We allowed the interior OA/oleate/POPC/POPE lipids to self-assemble spontaneously into vesicles by gradually raising the pH from 5.8 to 8.6 in the presence of 0.2 M ammonium acetate, which is a solute that is highly permeable through vesicle membranes^20,22,28^. Under these conditions, a pure OA/oleate/POPC/POPE mixture in 0.2 M ammonium acetate self-assembles into vesicles (control experiment in Supplementary Fig. 7 and Supplementary Note 4). The role of the ammonium acetate was to enable faster diffusion of buffer solutes and water into the interior of the plantosomes^28^, which was critical for plantosome expansion. In situ CLSM monitoring of plantosome expansion, including the proposed expansion mechanism, is presented in Fig. 5a–h (Supplementary Movie 2, transmission images in Supplementary Fig. 8). When the pH increased from 8.0 to 8.3, the interior of the plantosome was filled with vesicles (Fig. 5d). The largest expansion in plantosome size was observed in the last pH step^22^. The present uptake of water during expansion was not driven by the same osmosis mechanism as in plants: the concentration of buffer solutes (except for lipids) is adjusted quickly between the interior and exterior of the plantosomes at all time points during the pH increase due to the presence of ammonium acetate (see Supplementary Movie 2). Water uptake was achieved when the OA (in the interior of the plantosomes) converted to oleate with pH, and the oleate/OA/phospholipids self-assembled into vesicles (at pH > 8), as illustrated in Fig. 5g. The plantosome (Fig. 5a–f) increased 29% in radius that gives a surface area enlargement of 66%, which is somewhat larger than the values obtained for a larger population of plantosomes (*n* = 9): 13 ± 9% in radius increase and area increase of 28 ± 22%. In Fig. 5f, the same plantosome is shown after 1 h at pH 8.6 and demonstrate that the plantosome withstands the expansion over extended time periods. In Fig. 5i, j, the expansion of microcapsules (devoid of water cores), along with one plantosome, is presented. Such microcapsules (*n* = 4) were consistently enlarged to a much higher degree: in the given example, the average increase in radius was 68 ± 8% with an increase in the surface area of 184 ± 26%. The values obtained from a larger population of microcapsules (*n* = 22) were: 66 ± 12% and 177 ± 39% increase in radius and surface area, respectively (Fig. 5k, l). The larger expansion power is attributed to the high content of lipid in the interior; note that the capsule wall of microcapsules (with lipid only in the interior) also experience a large shrinkage during chloroform evaporation (Fig. 1d). In the latter case, the capsule wall demonstrated a remarkable extensibility. Previous studies showed that highly plasticized CNF/polysaccharide film can only be elongated in the order of 20% (at 20–40 wt% CNF) until it breaks^14^. Assuming only thinning (no necking) of the film, the area increase would be 20% for such a film, which is much lower than the observed area increase for the present microcapsule wall. Therefore, we hypothesize that the large expansion of the capsule surface cannot only be due to nanofiber pullout and capsule wall thinning, but is also enabled due to stretching a crumpled capsule wall surface. A crumpled/buckled surface area has previously been observed and reported for nanocellulose-based particles subjected to significant shrinkage during fabrication^29,30^. The structure arises at some point during the shrinking process, when the CNFs transit into a kinetically arrested state^10,11^. When this occurs, the surface buckles and a crumpled capsule wall structure, with folds on the submicron length scale, is obtained^30^. The exact morphology of the crumpled structure will depend on the shrinkage conditions (examples of crumpled capsules, after chloroform evaporation, are included in Supplementary Fig. 3a and 5, Supplementary Movie 1)^10,11^.

In all cases, the expansion resulted in a highly crowded interior lipid milieu (illustrated in Fig. 5g). The present vesicles were, however, too small to be observed with CLSM (CLSM images of larger vesicles, formed by fusing these small vesicles, are shown in the next section). After expansion, all expanded structures appeared similar, irrespective of the starting structure, i.e., a plantosome or a lipid-filled microcapsules (Fig. 5i, j). Therefore, we refer to all of them as expanded plantosomes. All the formed lipid compartments of the expanded plantosomes were highly permeable to 4 kDa FITC–dextran (Supplementary Fig. 9, Supplementary Note 5), due to the presence of 0.2 M ammonium acetate that is known to enhance the permeability of vesicle membranes^28^. The absence of Maltese crosses in the interior of expanded plantosomes (Supplementary Fig. 10, Supplementary Note 6) signified that the formed lamellar structures were thin and below the detection limit of an ordinary POM. Interestingly, the strong capsule wall, CNF, and pectin, did not burst (in most cases), taking on a similar role to that of the plant cell wall in real plant cells, although pores were present in the expanded wall (Fig. 4c–e, Supplementary Fig. 6), through which the lipids could escape. The primary cell wall in parenchyma cells still withstands high turgor pressures during the growth of the plant cells, despite being in a highly hydrated state, which is primarily due to the skeletal CNF network in the cell wall^13^.

**Fig. 6:**
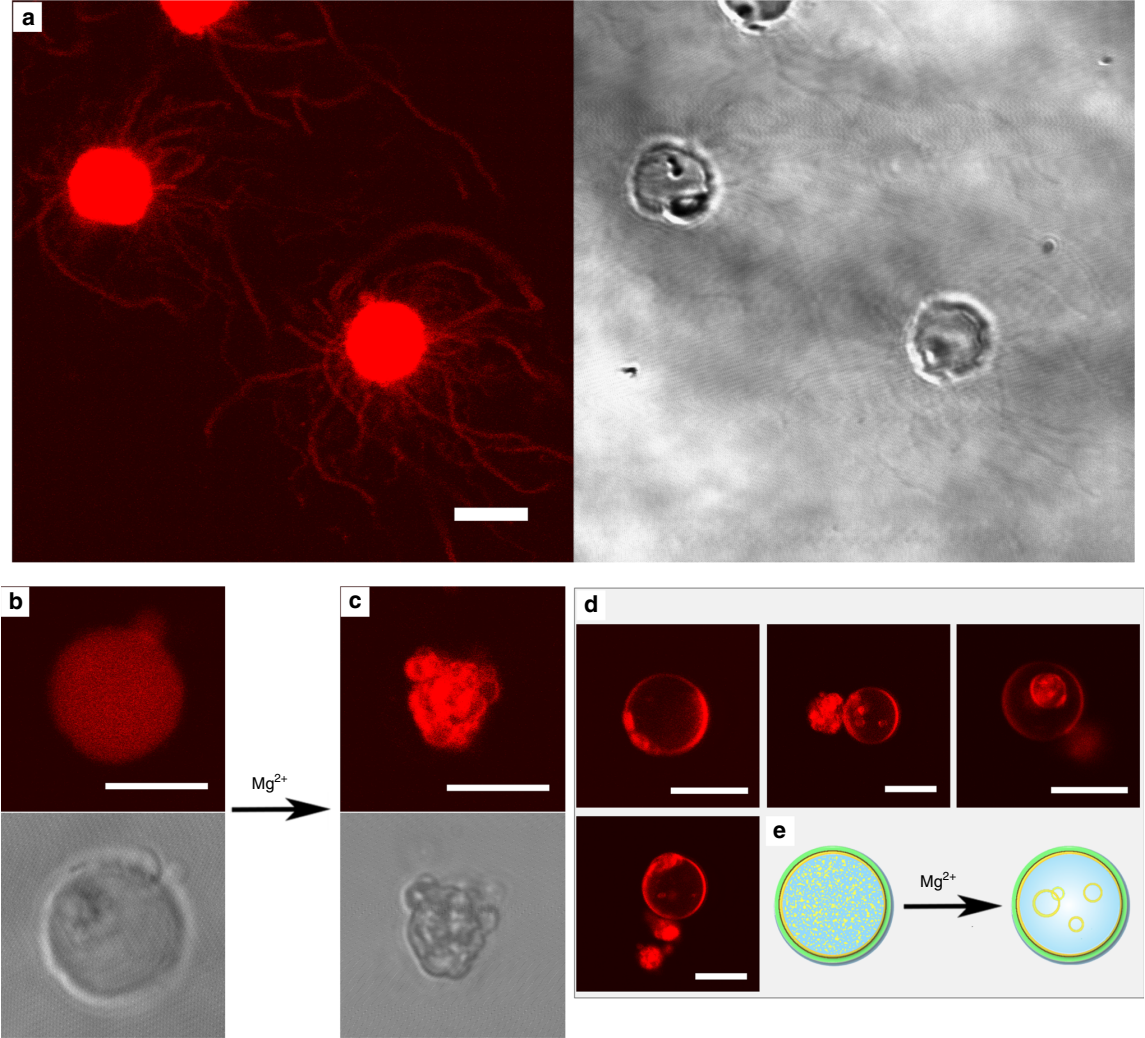
Lipid tubular structure formation and expanded plantosomes after exposure to Mg^2+^ ions. CLSM images (fluorescence and transmission images) of **a** lipid tubular protrusions from expanded plantosomes. Lipid tubular protrusions were observed in ~80% of the expanded plantosomes (*n* = 102, obtained from eight experiments). Several, but not all, expanded plantosomes collapsed in the presence of Mg^2+^: **b** an expanded plantosome at pH 8.6 and **c** after exposure to Mg^2+^ (image taken at 10 mM). In some instances, the interior lipids were rearranged: **d** plantosomes with (occasional) giant vesicles in the interior (at 10 mM Mg^2+^). Images in **b**–**d** are representative of two experiments. **e** Schematic representation of the fusion of small vesicles into larger vesicles and a continuous bilayer(s) at the plantosome inner interface that occurred for structures presented in **d**. Scale bars: 10 µm.

To release the pressure in some of the most overcrowded expanded plantosomes, tubular protrusions appeared and extended from the plantosome surfaces (Fig. 6a, faintly observed in Fig. 5j, Supplementary Movie 3, Supplementary Fig. 11, Supplementary Note 7). These protrusions remained attached to the expanded plantosome surface throughout the duration of the experiment. All of the plantosomes and lipid-filled microcapsules expanded during the pH increase (to 8.6), however, in some cases the CNF/pectin walls burst during expansion (10% of an expanded plantosome population of *n* = 116), which led to a halting of further expansion (Supplementary Fig.11b). For these, tubular protrusions were not observed (Supplementary Fig. 11b). But when the CNF/pectin wall did not burst, almost all of the expanded plantosomes exhibited tubular protrusions (we estimate that to be ~80%, *n* = 102), but a larger numbers of protrusions were observed from the surface of an expanded plantosomes that had completely been filled with lipid prior to expansion (e.g., a previous microcapsule devoid of a water core). OA/oleate vesicles have previously been reported in literature to grow as thread-like/tubular vesicles that are predisposed to divide^28,31^. These were created by feeding multilamellar OA/oleate vesicles with additional micelles to the exterior of the vesicles, and as the additional lipids were incorporated much faster into the outermost lipid membrane layer compared to the inner, the outermost membrane grew by forming protrusions^28^. An additional factor in the formation process was volume conservation between the lipid membranes in the multilamellar OA/oleate vesicle structure, due to the slow permeability of buffer solutes into the space between lipid membranes. However, in the presence of 0.2 M ammonium acetate, the permeability was increased and the same group observed that the outermost membrane layer of a multilamellar OA/oleate vesicle expanded as a sphere in the presence of supplementary exterior micelles^28^. Herein, we observe thread-like structures, in the presence of 0.2 M ammonium acetate. Moreover, extra micelles were not added to the exterior in the present experiments, and thus the source for additional lipids could not be other than the interior of the microcapsules/plantosomes. During expansion, the net pressure is exerted in the radial direction, and the lipid molecules will move from the interior toward the outer boundaries of the microcapsule/plantosome. We hypothesize that our lipid tubular structures are a consequence of the strongly restraining cage-like CNF/pectin wall, which limits the indefinite expansion and only allows the lipid to escape through holes in the CNF/pectin wall (see pores in the shell of an expanded plantosomes in Supplementary Fig. 6). To further prove this, we mixed the same lipid composition with an ammonium acetate solution, but in the absence of the CNF and pectin. The resulting vesicles were only spherical in shape (control experiment shown in Supplementary Fig. 7). Studies on artificial lipid membranes (GUVs) based on POPC and Rh-DOPE, that contained a crowded and viscous (protein) interior, have shown to deform in a similar manner as reported herein, i.e., via tubing deformations extending from their surfaces. This occurred, however, when the GUVs were placed in a hypertonic solution^32^. The excess membrane lipids (after shrinking) formed the tubular deformations and the initial viscosity inside the liposomes was the only determinant of the type of membrane deformation (tubular or bud deformations). We envision a similar mechanism for the present expanded plantosomes, which contain a crowded interior with excess lipids and a stiff CNF/pectin wall that imposes a constraining counter pressure during plantosome expansion, simulating that of the osmotic pressure of a hypertonic solution in the mentioned study^32^. However, to elucidate the exact formation steps for the present tubular structures, further investigations are needed. The present observation, however, implies that fatty-acid-based membranes with a small fraction of phospholipids have the ability to form tubular structures that extend beyond the boundaries of a rigid CNF/pectin cell wall, which has not been shown before. Pure fatty-acid-based membranes have been considered to be the earliest forms of cell membranes^19,33^ and the development of lipid tubular structures is pivotal for the development of hierarchical cellular structures in general and plant cells in particular. Indeed, today’s plant cells use tubular structures (up to hundreds of nm in diameter), called plasmodesmata, to connect neighboring cells across the cell wall in higher plant cells^12^. The plasmodesmata, which are connected to the endoplasmic reticulum (ER) in the interior of plant cells, are vital for intercellular communication, development and defense against pathogens^8,12,34^. These plasmodesmata are much more complex than the present lipid tubular structures^35^. However, model systems are the main tool used to study membrane properties and in particular membrane tubulation^36^. Even though the expanded plantosomes reported here are simple, they open up possibilities for deriving, and studying the structure and properties of highly curved lipid membranes in a plant cell model system.

Divalent ions, such as Mg^2+^, are known to bridge and fuse small vesicles into larger vesicles^16^. In an attempt to reduce the internal pressure in the expanded plantosomes, we exposed them to magnesium ions. The concentration of Mg^2+^ was increased in a step-wise manner, 2, 5, and 10 mM. In most cases, the expanded plantosome structure collapsed (Fig. 6b, c) sometimes already in the presence of 2 mM Mg^2+^ (Supplementary Movie 4). However, in some cases, the interior vesicles were transformed into a continuous bilayer(s) at the plantosome inner interface, in a similar manner as observed for other systems^16^. These plantosomes could then withstand higher Mg^2+^ concentration up to 5 mM (Supplementary Movie 4) or 10 mM, (Fig. 6d). In all of the cases, the tubular protrusions disappeared, suggesting that divalent ions changed the lipid packing in the plantosome membrane. Some interior vesicles also merged into giant vesicles that remained freely moving in the interior of the water-filled cavity (Fig, 6d, Supplementary Movie 4, schematic representation in Fig. 6e).

In conclusion, a strategy has been developed that overcomes the fundamental difficulties associated with assembling lipid vesicles with CNFs, which represents an important step toward advanced synthetic plant cells. The approach brings together different plant mimicking features and integrates lipid-mediated internalized structuration, expansion, and lipid tubular structure growth in the same microcapsule structures. Just like in natural parenchyma cells, our studies show the pivotal role that the CNF network has on restraining the expansion power of the interior aqueous environment. With our model, we show that a primitive fatty-acid-based membrane indeed can form tubular structures that stretch across a plant cell wall and we hypothesize that the cage-like plant cell wall and turgor pressure might have played an important role in the tubulation formation in primitive plant cells. The development of lipid tubular structures is a critical aspect of cell communication and the evolution of more hierarchical plant structures. The presented fabrication method opens up for future studies on more advanced plant cell models, permeability properties through plant cell wall/plasma membrane and intercellular ER models in different physiologically relevant settings and greater understanding of the evolution of plant cells. Additionally, this basic model could be further improved by increasing the complexity to answer the questions related to plasmolysis, ion responsiveness, and pH dependence of cell expansion. Finally, the fabrication protocol of plantosomes is useful, not only for model plant cells, but also in the production of model systems for algae, yeast, and bacteria.

## Methods

### Materials

Cellulose nanofibers (CNFs) modified with cationic quaternary ammonium groups (1.17 mmol g^−1^ fiber) was a kind gift (KTH, WWSC, Sweden). The CNFs were derived from never-dried softwood pulp (Nordic Paper Seffle AB, Sweden) and the reaction with glycidyltrimethylammonium chloride and homogenization steps were performed as described in detail earlier^37^. The CNFs have a high aspect ratio: the height is 2.5 ± 0.8 nm, obtained from atomic force microscopy (AFM) height measurements, and length is in micrometer range (Fig. 1a). The sugar beet pectin is a high methyl ester and naturally acetylated pectin (Genu®BETA-pectin) extracted from sugar beet pulp. It was a kind gift from CP Kelco (Lille Skensved, Denmark). The pectin has a degree of esterification ~55% and a degree of acetylation above 20%. OA (purity ≥99%), sodium chloride, chloroform (HiPerSolv ≥99.8%), Rh-6G, SR-101, calcofluor-white stain stock solution (contains calcofluor-white M2R, 1 mg mL^−1^ and Evans blue 0.5 mg mL^−1^), sodium hydroxide, hydrochloric acid, POPC, POPE, Rh-DOPE, FITC–dextran (4 kDa, FITC:glucose = 1:250), were all purchased from Sigma-Aldrich (Sweden). Ammonium acetate (97%) was purchased from Alfa Aesar GmbH (Germany) and ammonia solution (25%) was purchased from Merck. µ-Slides (four well) was purchased from Ibidi GmbH (Germany). All chemicals were used without further purification.

### Preparation of working and stock solutions

All working solutions were prepared just prior to the experiments. A suspension of CNF (0.059 wt%) in 100 mM NaCl was prepared by diluting a concentrated CNF suspension (0.39 wt%) with MilliQ-water and adding NaCl to obtain 100 mM NaCl. Afterward, the CNF suspension was magnetically stirred overnight (350 rpm) at room temperature (RT). The pH of the final suspension was 7.0 ± 0.2. To prepare 50 mL of the 0.2 wt% sugar beet pectin solution, 106 mg of pectin was dissolved in MilliQ water in the presence of ca. 180 µl of 1 M NaOH solution and magnetically stirred overnight at RT. The pH of the obtained solution was ca. 6.8. A 288 mM OA in chloroform solution was prepared by mixing 0.94 g of OA with 15.64 g chloroform. The solution was stored at 4 °C prior to use. The POPC/POPE phospholipid stock solution was prepared by mixing POPC and POPE in a molar ratio of 5:1 in chloroform. The final concentration was 1 mM POPC and 0.2 mM POPE in chloroform and it was stored at −20 °C. The Rh-DOPE/POPC/POPE phospholipid stock solution was prepared by mixing the fluorescently labeled phospholipid (Rh-DOPE) with POPC and POPE, the final concentration was 1 mM POPC, 0.14 mM POPE, and 0.09 mM Rh-DOPE. The stock solution was stored at −20 °C. Prior to the experiments all stock solutions were brought to RT.

### Preparation of microcapsules

From the working solutions, 1.5 g of CNF suspension (0.059 wt% CNF in 100 mM NaCl, pH 7 ± 0.2) and 2.23 g of OA in chloroform (288 mM) was added to a 15 mL Falcon tube (Supplementary Fig. 1a). The CNF-stabilized lipid droplets were obtained by mixing with an IKA T25 digital Ultra Turrax (24,000 rpm), Supplementary Fig. 1b. The mixing was carried out in a sequential way, i.e., 15 s mixing and 10 s pause, repeated three times, to ensure extensive mixing. After that, the droplets were allowed to settle for ~ 15 min prior to use. They phase separated into the upper water phase, middle CNF-stabilized oil droplets and lower chloroform phase, Supplementary Fig. 1c. The droplets, obtained from the middle part of the Falcon tube in Supplementary Fig. 1c, were immediately utilized to prepare microcapsules.

Equal amounts of the 0.2 wt% pectin working solution and a 200 mM NaCl in MilliQ-water solution (10 g each) were mixed (in a 40 mL glass vial) and the pH was adjusted to 10.0 with a 1 M or 0.1 M NaOH solution. The final working concentration of the pectin solution was 0.1 wt% in 100 mM NaCl. An amount of 300 µL of the CNF-stabilized OA/chloroform droplets (middle phase, Supplementary Fig. 1c) was carefully taken and transferred into the pectin in 100 mM NaCl solution and the chloroform was evaporated under magnetic stirring (350 rpm) overnight (15–18 h). Though the initial pH of the pectin was 10.0, immediately after adding the CNF-stabilized oil droplets to the pectin solution, the pH of the dispersion was continuously dropping down during evaporation and stabilized at around pH 6.1. This is a consequence of the dissociation of OA into oleate in the presence of salt and/or higher pHs^20^. After evaporation, the microcapsules float to the surface of the solution due to the entrapped OA/oleate. Then, the pH of the solution was adjusted to 2.0 with 100 mM HCl, and after 30 min, the pH was raised to 6.5 with 100 mM NaOH to obtain the final microcapsules. Supplementary Fig. 3c, d shows the overall transformation of CNF-stabilized lipid droplets into microcapsules, as derived from the microscopy images in Supplementary Fig. 3a. The microcapsule suspensions were stored at RT prior to further characterization.

### Preparation of plantosomes

An amount of 0.5 mL of the POPC/POPE stock solution (1 mM/0.2 mM POPC/POPE), 0.5 mL of chloroform, 1 mL of OA (288 mM stock solution), and 2.3 g of the CNF suspension (0.059 wt% CNF in 100 mM NaCl, pH 7 ± 0.2) was added into a 15 mL Falcon tube and mixed an IKA T25 digital Ultra Turrax (24,000 rpm, 15 s mixing, 10 s pause, three repetitions). When the droplets also contained Rh-DOPE, 40 µL of the Rh-DOPE/POPC/POPE phospholipid stock solution was included in the above mixture and 0.48 mL of the POPC/POPE stock solution was used instead of 0.5 mL. The droplets were allowed to settle for ~15 min prior to use, see Supplementary Fig. 1f. A light microscopy image of the obtained droplet is found in Fig. 3a, which shows that some of them contained very large water cavities in the interior. Droplets, obtained from the middle part of the Falcon tube, were collected.

These CNF-stabilized OA/phospholipid/chloroform droplets (taken from the middle phase, see Supplementary Fig. 1f) could not be directly transferred to a 0.1 wt% pectin in 100 mM NaCl solution (pH 6.3), because aggregates were observed. To circumvent this, 300 µL of the CNF-stabilized OA/phospholipid/chloroform droplets was first dispersed in 1.5 mL Eppendorf tube containing 0.7 g of the 200 mM NaCl in MilliQ-water solution. The droplets were dispersed by repeatedly (trice) sucking and releasing the suspension with a Pasteur pipette, followed by transferring the dispersion to 9.3 g of 200 mM NaCl in MilliQ-water solution. Afterward, the suspension was transferred to the 0.2 wt% pectin solution (10 g, pH 6.8) present in a 40 mL glass vial. Now the droplets were in a 0.1 wt% pectin in 100 mM NaCl solution with pH 6.3. The chloroform was evaporated under magnetic stirring (350 rpm) overnight (15–18 h). The pH of the solution dropped (due to deprotonation of OA) during evaporation and stabilized at around pH 5.8–5.9. The tubes and glass vials were protected from light using aluminum foil when Rh-DOPE was present. The final suspension was a mixture of capsules with large water-filled cavities (plantosomes), and in some cases, the water cavity was missing (microcapsules), see Fig. 3b. The plantosomes yield was 44 ± 21 % (calculated from the number of plantosomes prior to and after chloroform evaporation). The suspensions were stored at RT prior to further experiments.

### Light microscopy

Microcapsules and plantosomes, both during preparation and final structures (Fig. 1e, f, Fig. 3a, b, Supplementary Figs. 3a and 5), were studied with upright light microscope (VisiScope, VWR, Sweden) equipped with VisiCam 16 Plus camera (IS VisiCam Image Analyser 3.9.0.605 software), and 40× and 20× air objectives. Plantosomes were also studied using an Axio Vert.A1 Light Microscope (Carl Zeiss, Germany) equipped with a Zeiss AxioCam 305 color camera (Zeiss Zen 2.6 (blue edition) software), and 20× and 40× air objectives. The size of microcapsules and plantosomes, at different conditions, were measured with Image J 1.50b (NIH, USA) or Zeiss Zen 2.6 (blue edition).

To calculate the plantosome yield, 100 µL of suspension was dropped on a microscopic slide and covered with a coverslip and the number of plantosomes in the suspension were calculated from 20 images (151 ≥ *n* ≥ 35), which were randomly taken (20× objective, Axio Vert.A1 Light Microscope, Zeiss Zen 2.6 (blue edition)). The yield was calculated from the number of plantosomes prior to and after chloroform evaporation. The yield is an average from five separate experiments (yields 26%, 38%, 30%, 45%, and 80%).

### Polarized optical microscopy

The organization of lipids in the interior of the microcapsules, plantosomes, and expanded plantosomes was studied using an inverted Axio Vert.A1 Light Microscope (Carl Zeiss, Germany, Zeiss Zen 2.6 (blue edition) software) equipped with cross-polarized light filters and with 10×, 20×, and 40× air objectives and a Zeiss AxioCam 305 color camera.

POM was used to monitor the change of molecular organization of OA/oleate inside microcapsules (microcapsule with only OA/oleate cores) in acidic and alkaline conditions, as well as the lipid release from microcapsule interior and microcapsule wall expansion. The results presented in Supplementary Movie 1. A 100 µL microcapsule suspension, containing crumpled microcapsule structures obtained after chloroform evaporation, see light microscopy image in Supplementary Fig. 3a, after evaporation, was dropped on a microscopic slide and covered with a coverslip. The experimental setup is shown in Supplementary Fig. 12. A 100 mM HCl solution was added at the rate of 2 µL min^−1^ using a syringe pump (New Era Pump systems, USA), until the microcapsule appeared spherical in shape. Afterward, a 100 mM NaOH was added at the same rate to observe lipid release and microcapsule expansion. The empty microcapsule walls were stained with calcofluor-white stain stock solution (1.5 mg mL^−1^), with the same pumping rate. After the dye reached the observation point, the flow was stopped and different capsules were imaged.

### Atomic force microscopy

CNF (0.05 wt%) was dispersed in water and stirred overnight (350 rpm) at RT then the CNF was sonicated with Sonics Vibra-Cell, 80% amplitude, 750 W, 1/2″ tip for 60 s, (30 s on, 10 s off). Then the CNF was further diluted with water and 0.0025 wt% CNF was obtained. Plasma activated clean silicon wafer was dipped (1 min) in the CNF suspension and dried with nitrogen and AFM images were obtained (Scanasyst-air cantilever) using Bruker NanoScope V (U.S.A.). Images of 2 × 2 µm in size with 512 × 512-pixel resolution were recorded using the software NanoScope 8.15. The images were analyzed using Gwyddion 2.47.

### Quartz crystal microbalance with dissipation

The interaction of CNF and pectin was examined by Quartz crystal microbalance with dissipation (QCM-D), model E4, (Q-Sense, Sweden) using Au-coated quartz crystals. Before the experiments, the crystals were thoroughly cleaned with the procedure described earlier^38^ and the adsorption steps were carried out at 25 °C with a constant flow rate of 100 μL min^−1^. The QCM-D results for CNF (0.059 wt% in 100 mM NaCl, pH 7 ± 0.2) and pectin (0.1 wt% pectin in 100 mM NaCl, pH 6.3) interaction and only the adsorption of pectin on the Au-sensor is shown in Supplementary Fig. 4c. Prior to and after the adsorption of CNF or pectin, a washing step was included using 100 mM NaCl in MilliQ-water. Sufficient time was allowed until a steady QCM-D signal was attained for each step.

### Cryo-transmission electron microscopy

Samples were imaged with a JEM-2100f (Jeol Ltd., Japan). A drop of the suspension containing vesicles was added to a Quantifoil holey carbon grid (R2/2, 200 mesh) and plunge-frozen (FEI Vitrobot Mark III). The vesicle suspension was prepared almost in the same way as plantosomes are prepared, but in the absence of CNF and pectin. A droplet of the lipid in chloroform (consisting of 173 µL of 288 mM OA stock solution, 83 µL of POPC/POPE stock solution, and 6.92 µL of the Rh-DOPE/POPC/POPE phospholipid stock solution) was added to 20 g of a 100 mM NaCl solution in MilliQ-water (pH ca. 6). The solution was put on magnetic stirring (350 rpm) overnight (17 h) to allow the chloroform to evaporate. The following day, 20 g of a solution consisting of 0.4 M ammonium acetate and 100 mM NaCl solution in MilliQ-water (pH 8.62, adjusted with ammonia) was added. The suspension was shaken by hand and the pH was adjusted to 8.6 with ammonia. Afterward, the suspension was passed six times through a 0.2 µm syringe filter. The lipid concentration in the final suspension was 1.2 mM.

### Transmission electron microscopy

High-magnification transmission electron micrographs of the microcapsules/plantosomes were acquired using a TEM from Hitachi, model HT7700 (Japan, Hitachi HT7700 02.05 software) at an accelerating voltage of 100 kV in high-contrast mode. The microcapsules/plantosomes (after chloroform evaporation) were first washed with water (suspension diluted 20 times with MilliQ-water, separation of microcapsules/plantosomes from water phase. Taking 200 µL of the separated microcapsules/plantosomes and diluting with 800 µL MilliQ) and deposited onto 200 mesh Formvar/carbon TEM grids (Ted Pella, 01800-F) and thoroughly air-dried. Then the lipid contents were removed by dipping the TEM grid into 2.5 mL of 100 mM NaOH solution for 3 min followed by thorough washing with MilliQ water (dipping into 2.5 mL MilliQ-water for 2 min and repeating a second time with fresh MilliQ-water) and air-dried before imaging. The images were taken without staining.

### Scanning electron microscopy

After TEM analysis, the same samples (on TEM grids) were grounded with Pt/Pd (60/40) for 20 s at a current of 80 mA using a Cressington 208HR sputter coater and high-resolution scanning electron micrographs were acquired using a SEM from Hitachi, model S-4800 (Japan, S-4800 04.05 software) at an accelerating voltage of 1 kV (Supplementary Fig. 6a, b).

### Confocal laser scanning microscopy

CLSM imaging was performed using a Zeiss 780 UV/Vis (Zeiss, Germany, Zen Black 2012 software) equipped with C-Apichromat 40 × /1.2 NA water immersion objectives at RT (Figs. 2a–d, 3d–f and 4a, b, f in the main manuscript) or a LSM 510 UV/Vis (Zeiss, Germany, LSM 510 3.2 SP2 software) equipped with 40 × /1.3 NA air objectives (Figs. 5 and 6, Supplementary Figs. 8, 9 and 11). Just prior to the experiments, the fluorophores Rh-6G (lipophilic dye^23^, see Supplementary Note 8), SR-101 (hydrophilic dye), and 4 kDa FITC–dextran with concentrations 0.02, 1.0, and 2 mg mL^−1^, respectively, were prepared in 100 mM NaCl and the pH was adjusted to 6.5 (Figs. 2, 3 and 4f). The outer encasing CNF-rich plantosome wall was revealed by exposing the plantosomes to cellulose-specific calcofluor-white stain (stock solution 1.5 mg mL^−1^). An equal volume of the freshly prepared fluorophores and the microcapsule (or plantosome) suspension were mixed and the microcapsules were studied with CLSM.

The permeability of 4 kDa FITC–dextran through expanded plantosomes (that also contained Rh-DOPE, results in Supplementary Fig. 9) was studied using 4 kDa FITC–dextran (stokes radius of ~1.4 nm, producer’s information) at a concentration of 1 mg mL^−1^ prepared in 0.2 M ammonium acetate, 100 mM NaCl, pH 8.8 or 8.7 (adjusted with ammonia). Details of the experimental setup used to expand and study the plantosomes are given in the next section. The laser excitation wavelengths used were 514 nm (Rh-6G), 561 nm (SR-101), 488 nm (FITC–dextran), 561 or 543 nm (Rh-DOPE), and 405 nm (calcofluor-white). Images were analyzed with Zeiss Zen 2.6 (blue edition) or Zeiss LSM Image Browser.

### Experimental setup for in situ monitoring with CLSM

The effect of different pH values and MgCl_2_ concentrations was studied (in the presence of ammonium acetate and NaCl) by using the plantosomes/microcapsule/expanded plantosomes with Rh-DOPE and CLSM. Details of the CLSM instruments and objectives used are found in the previous section. To study the effect of pH, 0.2 M ammonium acetate was prepared in 0.1 M NaCl solution and the pH was adjusted to 6.5, 8.2, 8.4, and 8.7–8.8 (with ammonia). A 0.4 M ammonium acetate in 0.1 M NaCl solution, pH 6.5, was also prepared. Different concentrations of MgCl_2_ (2, 5, and 10 mM) were prepared in a solution composed of 0.1 M NaCl and 0.2 M ammonium acetate, and the pH was adjusted to 8.7 or 8.8 (with ammonia). In situ observations were performed using a modified µ-slide well (Ibidi, Germany), as shown in Supplementary Fig. 13. The spacer, cover-slide, and tubes were glued using thiol resin. First 100 µL of capsule suspension (plantosomes and microcapsules) was added, and plantosomes/capsules entered the space between the cover slides (space ca. 400 µm in height) due to capillary forces. Then 500 µL of a 0.1 M NaCl solution in MilliQ water was added. The setup was placed in the microscope and the tubes were connected to pumps and the area of interest was located. Then 500 µL of 0.2 M ammonium acetate solution (pH 6.5) followed by 500 µL of 0.4 M ammonium acetate solution (pH 6.5) was added carefully with the pipette and equilibrated for ~1 h. Now the surrounding medium was a 0.19 M ammonium acetate, 0.1 M NaCl, pH ~6.5. After that the 0.2 M ammonium acetate solutions with different pH (8.2, 8.4, and 8.7 or 8.8) were pumped (using syringe pump) one by one at the rate of 200 µL min^−1^ and the solutions was pumped out (using a peristaltic pump) from the chamber at the same speed and collected (Supplementary Fig. 13). For the collected liquid (1.5 mL aliquot) at the outlet, the pH was recorded and reported. During the final pH increase (from 8.3 to 8.6), the targeted pH was set to 8.60–8.69, and either a solution with pH 8.7 or 8.8 was used to reach this final pH. Results are found in Figs. 5 and 6a, Supplementary Movie 2 and 3, Supplementary Figs. 8, 9 and 11. After that, MgCl_2_ solutions (2, 5, and 10 mM) was pumped (200 µL min^−1^) and the changes were recorded (results in Fig. 6b–d and Supplementary Movie 4). Here, the pH was also set to a pH between 8.60 and 8.69, and to achieve this, MgCl_2_ solutions (composition described above) with either pH 8.7 or 8.8 was used. Each solution was pumped for 45–60 min.

### Reporting summary

Further information on research design is available in the Nature Research Reporting Summary linked to this article.

## Supplementary information

Supplementary Information file

Reporting summary

Description of Additional Supplementary Files

Supplemenatary Movie 1

Supplemenatary Movie 2

Supplemenatary Movie 3

Supplemenatary Movie 4

## Data Availability

Data supporting the main findings of this work are available within the paper and its Supplementary Information files. A reporting summary for this Article is available as a Supplementary Information file. Additional data generated and analyzed during the current study are available from the corresponding author upon request. The source data underlying Fig. 5k, l are provided as a Source Data file.

## Source Data

Source Data file
