# Supplementary material for: Primary cell wall inspired micro containers as a step towards a synthetic plant cell
Source: Nat Commun. 2020 Feb 19;11:958. doi: 10.1038/s41467-020-14718-x (PMC7031234; doi:10.1038/s41467-020-14718-x)
Supplement: Supplementary file 1 — Supplementary Information file [file 41467_2020_14718_MOESM1_ESM.pdf]

# Primary cell wall inspired micro containers as a step towards a synthetic plant cell

Paulraj *et al.*

## Supplementary Method 1

### Experimental details

The importance of CNF in stabilizing the oil/water (O/W) interface (for all lipid composition mentioned above) could be understood from comparing with a system that was mixed with a 100 mM NaCl solution (pH 7.0) devoid of CNF, compare results in Supplementary Fig. 1 and Supplementary Fig. 2. No droplets were obtained in the absence of CNF, which clearly shows that CNF is very important in stabilizing O/W interface.

## Supplementary Method 2

### Assembly of Microcapsules with OA/oleate cores

As can be seen, after evaporation the microcapsules display a deflated/buckled shape (Supplementary Fig. 3a- after evaporation). The appearance of the microcapsules after chloroform evaporation highly depends on the initial pH of the surrounding pectin solution, see Supplementary Note 2. However, despite the deflated/buckled shapes, the lipids (and also small water droplets) were well retained within the core, very little free (or none) OA/oleate were observed after evaporation in the bulk**.** During the pH decrease to 2.0, the capsule shape transforms from a buckled structure to a more apparent spherical shape (Supplementary Fig. 3a-at pH 2.0, Supplementary Movie 1). No coalescence, flocculation or breaking/leaking was observed. The resulting capsules were 22 ± 11 µm in size. By increasing the pH to 6.5, a minor influence on the average capsule size, 27 ± 11 µm, was observed, results in Supplementary Fig. 3a-at pH 6.5.

## Supplementary Note 1

### The importance of pectin

The importance of the pectin during the preparation of the microcapsules with OA/oleate cores is illustrated in Supplementary Fig. 4a and 4b: in the absence of pectin the obtained material got stuck at the magnet (b), whereas microcapsules were well-dispersed (a) when pectin is present and adsorbed onto the microcapsules. QCM-D results for cationic CNF and pectin layer formation is presented in Supplementary Fig. 4c. An Au-sensor was used in the experiments and the pectin was able to adsorb directly onto the Au surface (see reference measurement in the same figure).

However, when CNF was first deposited (layer 1, Supplementary Fig. 4c), more pectin adsorbed (layer 2), which clearly demonstrates that pectin has affinity to CNF.

## Supplementary Note 2

### Effect of the initial pH on the resulting microcapsule morphology after chloroform evaporation

The initial pH of the pectin solution is also an important factor that affects the appearance of the obtained microcapsules and the distribution of lipids throughout the interior the encasing CNF/pectin shell. If the initial pH of the pectin solution was 10, a deflated/buckled morphology is observed in light microscopy (the pH of the microcapsule suspension after chloroform evaporation is 6.15, Supplementary Fig. 5a), where the lipids appear to be evenly spread throughout the interior. However, if a lower pH of the pectin solution was used (starting pH 6.1), then a more *apparent* spherical structure was observed in light microscopy (pH after evaporation was 5.7 Supplementary Fig. 5b). Our light microscopy observations, however, suggest that the lipids were present as spherical droplets in the interior whereas the CNF/pectin wall was folded around and present at a distance from the lipid droplets, see arrows in Supplementary Fig. 5b which point to a thin crumpled capsule wall that is located around an interior lipid droplet. OA forms oil droplets in water at low pH (<7).[1,2] Such a crumpled CNF/pectin wall can be expected, considering the very large amount of chloroform that evaporates during microcapsule formation. The crumpled wall most likely arises at some point during the microcapsule shrinking process, when the CNFs transit into a kinetically arrested state. [3,4]

## Supplementary Note 3

### SEM and TEM images of the enchasing CNF/Pectin shell in expanded plantosomes after lipid-core removal

The CNF/pectin wall of expanded plantosomes, which had been emptied of their interior lipid cores, were studied with SEM and TEM, the results are presented in Supplementary Fig. 6. In the case of TEM, a shell that burst during lipid release was selected, in order to better visualize the CNFs. The average size of the pores present in the CNF/pectin wall were 18 ± 12 nm (n=120, obtained from SEM images). Larger pores also appeared occasionally which were several tens of nm in diameter (black arrow in Supplementary Fig. 6b). It should be noted that the images are of

dried CNF/pectin walls of expanded plantosomes and that the attained pore-size might differ from those in the wet state.

## Supplementary Note 4

### OA/oleate/POPC/POPE mixture forms vesicles in buffer

The cryo-TEM micrograph in Supplementary Fig. 7 shows that a neat OA/oleate/POPC/POPE/Rh-DOPE mixture in 0.2 M ammonium acetate and 100 mM NaCl was able to assemble into vesicles at pH 8.6, in line with previous reports.[1,5,6] The lipid concentration was 1.2 mM. All generated vesicles were round in shape, but the cryo-TEM micrograph also showed that some of the vesicles had a tendency to aggregate. This has previously been reported in literature for OA/oleate vesicles.[5] The same authors showed that larger clusters were formed at pH close to 8.0, whereas less was observed as the pH increased to 9.00. However, they observed no increase in size with time despite the tight aggregation.[5] The present vesicles were stable up for at least 3 days as measured by dynamic light scattering.

## Supplementary Note 5

### Permeability properties

The expanded plantosomes in 0.2 M ammonium acetate were highly permeable to 4 kDa FITC-dextrans, Supplementary Fig. 9. The 4 kDa FITC-dextran penetrated into all compartments of the expanded plantosomes (both lipid tubular protrusions and interior) within seconds. The permeability experiments were done in the presence of ammonium acetate. Ammonium acetate is known to enhance the diffusivity of small molecules through vesicle membranes.[6]

## Supplementary Note 6

### POM of expanded plantosomes

The expanded plantosomes, at pH 8.6 and present in modified wells (Supplementary Figure 13), were imaged with POM to understand the organization of the lipids in the interior, see Supplementary Fig. 10a-b. Very few Maltese crosses were observed, see arrows in Supplementary

Fig. 10b, and their absence in most expanded plantosomes signified that the formed lamellar structures were thin and below the detection limit of an ordinary polarized optical microscope.

## Supplementary Note 7

### Tubular protrusion from surface of expanded plantosomes

Due to the inner pressure inside the expanded plantosomes, lipid started escaping out through the CNF/pectin walls in the form of elongated tubular protrusions (see white arrow in Supplementary Fig. 11a). The strong encasing CNF network structure played a pivotal role in this case. In some cases, however, the capsule wall also fractured and released the entire content (Supplementary Fig. 11b). Interestingly, in the latter case, all of the lipid content (OA/oleate, POPC, POPE and Rh-DOPE (red)) was always encased inside a spherical lipid membrane structure, the whole thing observed as a red dot, see white arrow in Supplementary Fig. 11b. Some lipid also remained adsorbed at the inner surface of the capsule wall, see green arrow in Supplementary Fig. 11b.

## Supplementary Note 8

### Solubility of Rhodamine 6G in the oil and water phase

The solubility of Rhodamine 6G (Rh-6G) in a mixture of water (containing salt) and oleic acid at pH 6.5 was investigated. The dye was dissolved in the aqueous phase (1 mg $mL^{-1}$, 9 mL solution) and salt (100 mM NaCl in the aqueous phase) and 500 µL oleic acid was added. The pH was adjusted using 0.1 M NaOH solution to 6.5, where-upon the dye was redistributed within the oil and water phases. The dye was soluble in both the oil and water phase, although the dye had a higher affinity to the oil phase. The amount of dye in the different phases was oil phase; 17.4 mg dye/g oil and in the water phase; 0.17 mg dye/g water. The amount of dye was obtained by fluorescence spectroscopy by analysing the amount of dye present in the water phase ($\lambda_{ex}$= 500 nm, $\lambda_{em}$= 554 nm, Microplate Reader Infinite M200PRO, TECAN). From this concentration, the amount of dye in the oil phase was obtained.

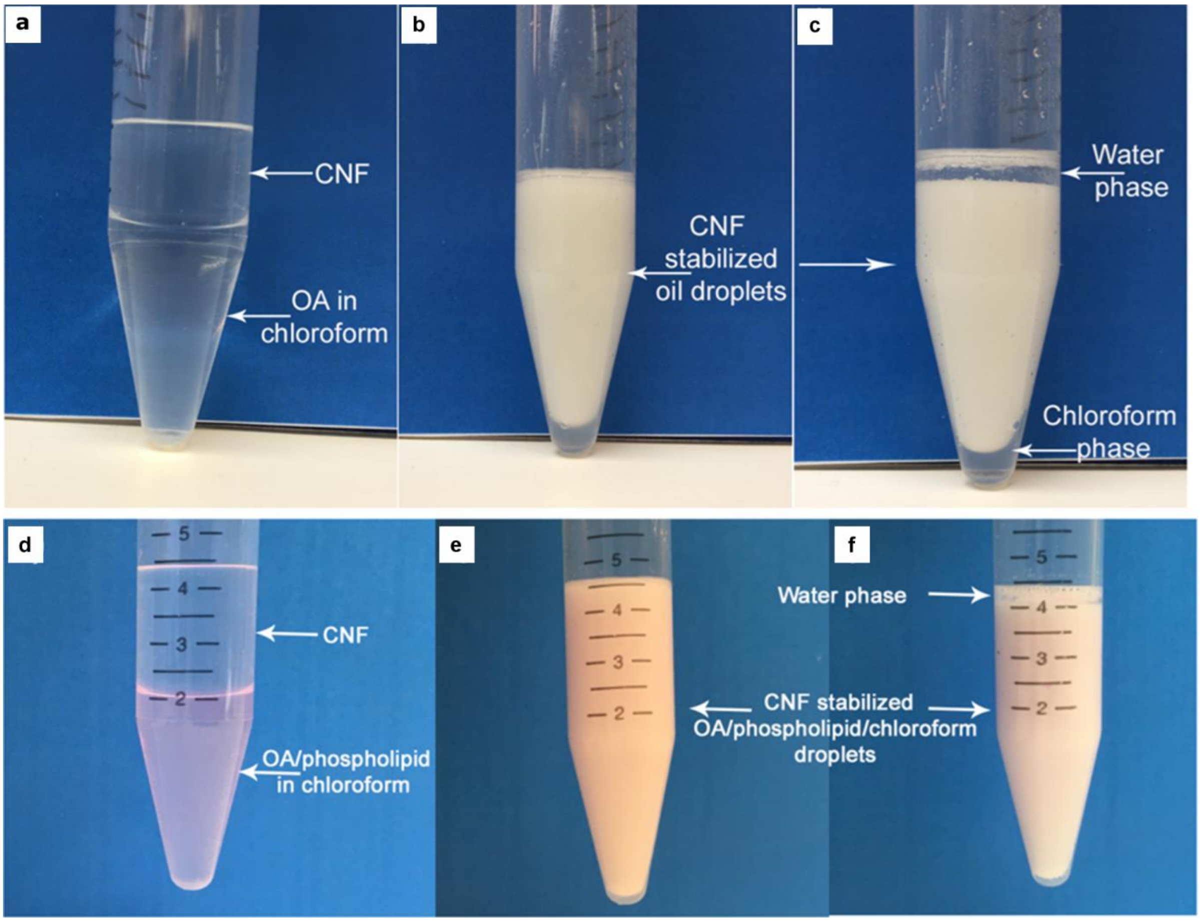


**Supplementary Figure 1. Photographic images of the preparation of CNF stabilized lipid droplets.** CNF (0.059 wt% in 100 mM NaCl, pH 7 ± 0.2), OA (288 mM) in chloroform: a) before mixing, b) immediately after mixing and c) after 15 min. Photographic images of the preparation of CNF stabilized OA/phospholipid/chloroform droplets. CNF, OA/phospholipids in chloroform: d) before mixing, e) immediately after mixing and f) after 15 min. The CNF stabilized oil droplets, taken from the middle phase in the Falcon tubes shown in c) and f), were used for microcapsules preparation.

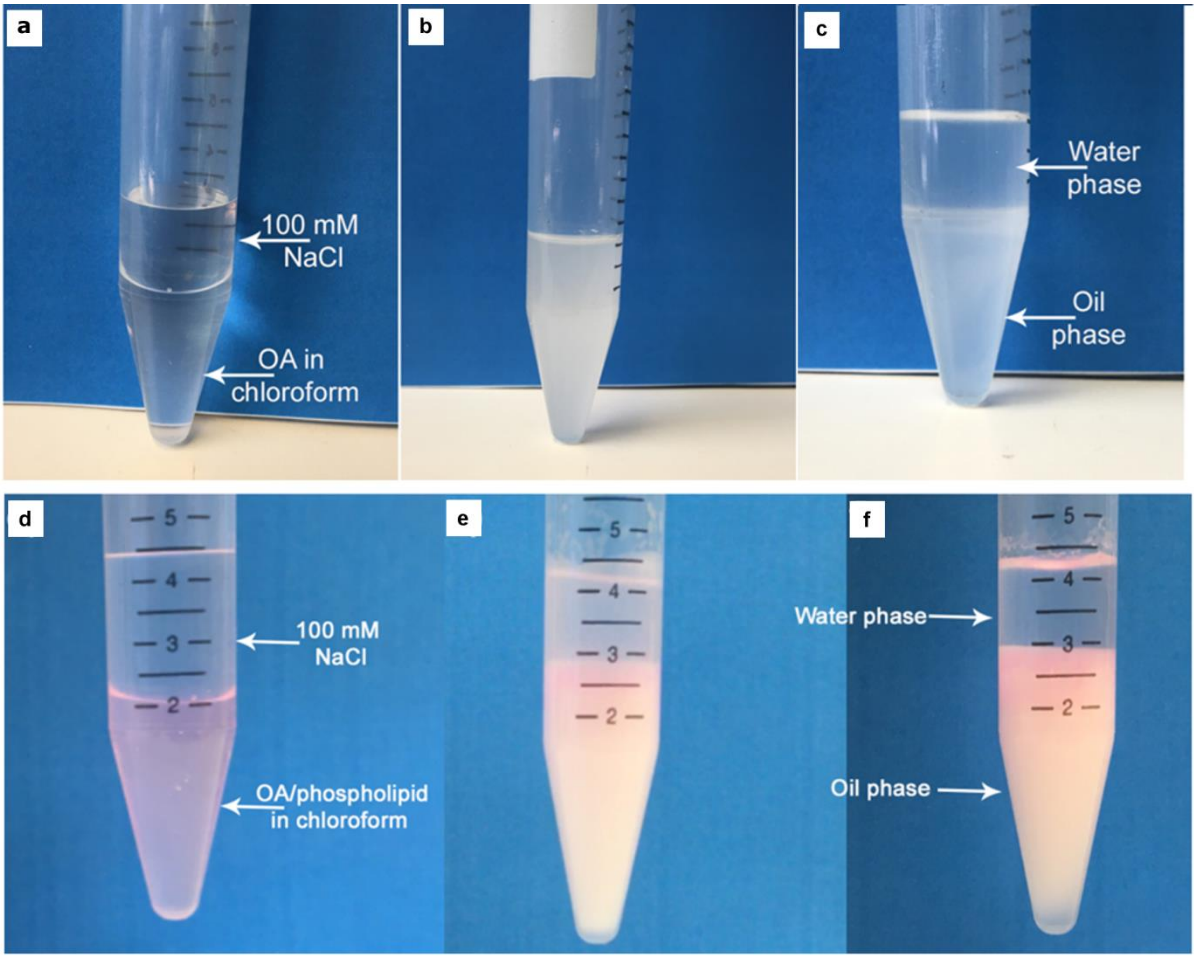


**Supplementary Figure 2. Lipid droplet formation requires CNF.** Photographic image of 100 mM NaCl (pH 7.0) and 288 mM OA in chloroform in absence of CNF: a) before mixing, b) immediately after mixing and c) after 15 min. Photographic image of 100 mM NaCl (pH 7.0; without CNF) and OA, phospholipids in chloroform: d) before mixing, e) immediately after mixing and f) after 15 min. Compare with results in Supplementary Fig. 1.

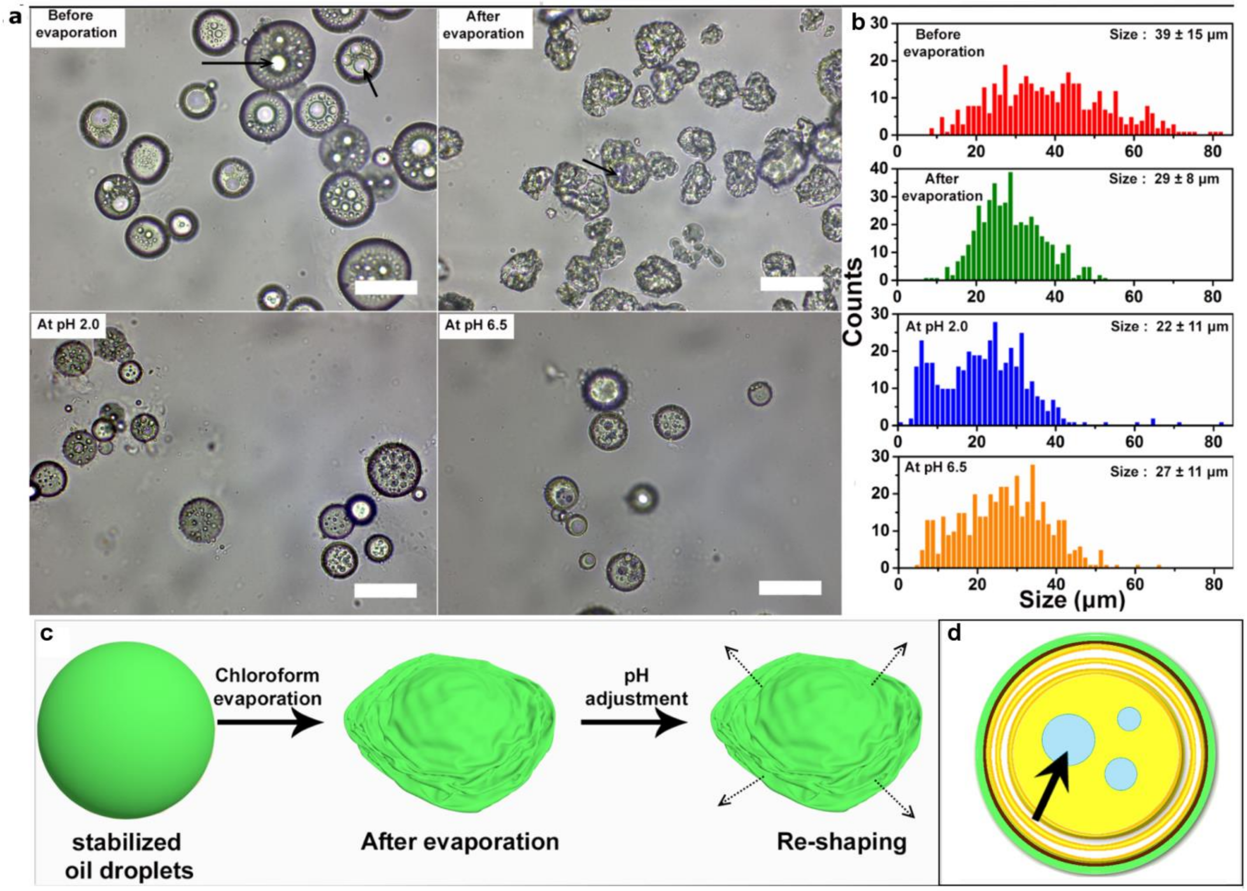


**Supplementary Figure 3. Preparation of the microcapsules with OA/oleate cores only.** (a) Light microscopy images of the microcapsules at different stages of the preparation: prior to chloroform evaporation, after chloroform evaporation, at pH 2.0 and pH 6.5 and the corresponding size distribution (b) determined by Light microscopy. Data in (b) was collected from four experiments, and histograms include n = 402 CNF/pectin stabilized oil droplets before evaporation, n = 455 after chloroform evaporation, n = 433 at pH 2.0 and n = 447 microcapsules (at pH 6.5). Schematic illustration (c) of the different steps in the microcapsule formation and (d) cross-section of the final microcapsules obtained. Green - pectin, brown - CNF, blue – water, yellow – lipid. Scale bars: 50 μm

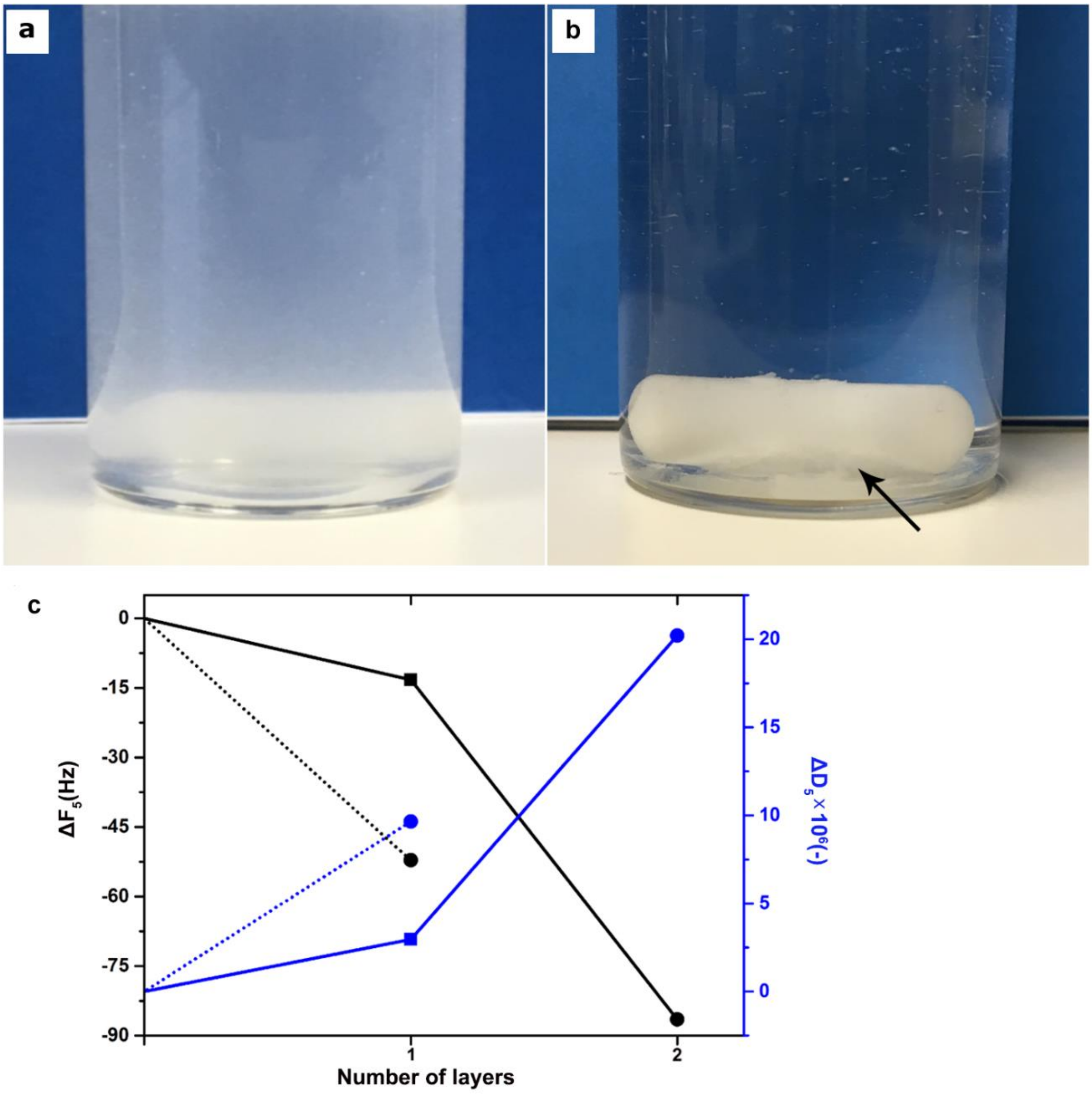


**Supplementary Figure 4. Importance of pectin.** Photographic images of CNF stabilized oil droplets (OA/oleate/chloroform) during chloroform evaporation in (a) the presence and (b) absence of pectin. In (b) only a 100 mM NaCl solution at pH 10.0 was used, whereas in (a) 0.1 wt% pectin in 100 mM NaCl at pH 10.0 was used. In the absence of pectin, a clear suspension was obtained and the arrow points to the formation of CNF aggregates around the magnetic stir bar. (c) QCM-D result showing the net changes in the frequency ($\Delta F_5$, black lines) and dissipation ($\Delta D_5$, blue lines) for the adsorption of cationic CNF followed by adsorption of pectin (solid lines). A reference measurement (dotted lines), showing direct adsorption of pectin onto the Au-sensor is also included. Pectin (black or blue circles) and CNF (black or blue squares). Data are for the fifth overtone.

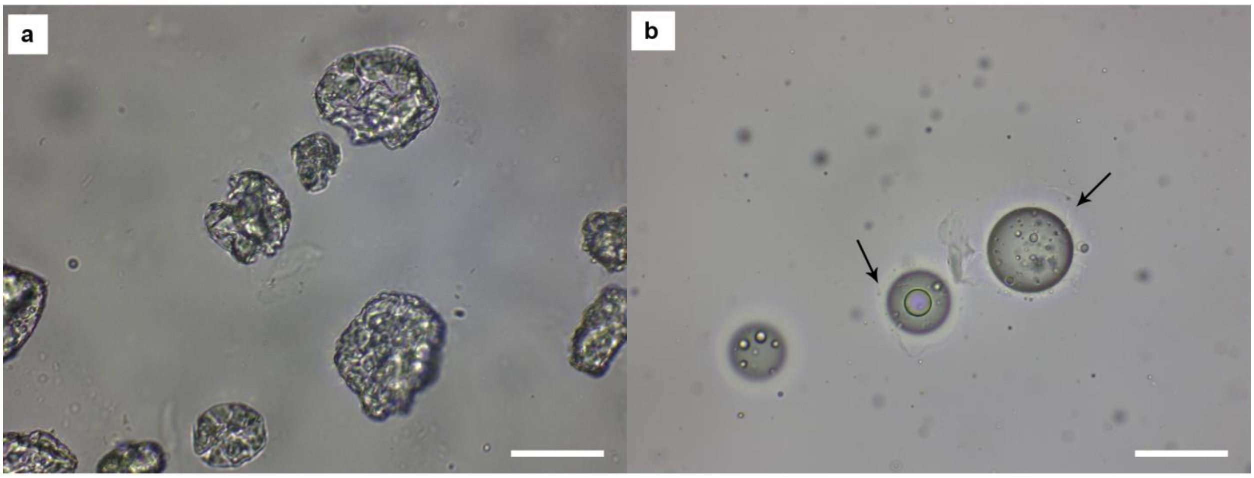


**Supplementary Figure 5. Resulting microcapsule morphology as a function of the pH of the pectin solution.** Bright field images of microcapsules with OA/oleate cores obtained after chloroform evaporation when (a) the initial pH of the pectin solution was 10 and (b) when the initial pH of the pectin solution was 6.1. Images in (a) and (b) are representative of eight and three experiments, respectively. Scale bars: 50 µm.

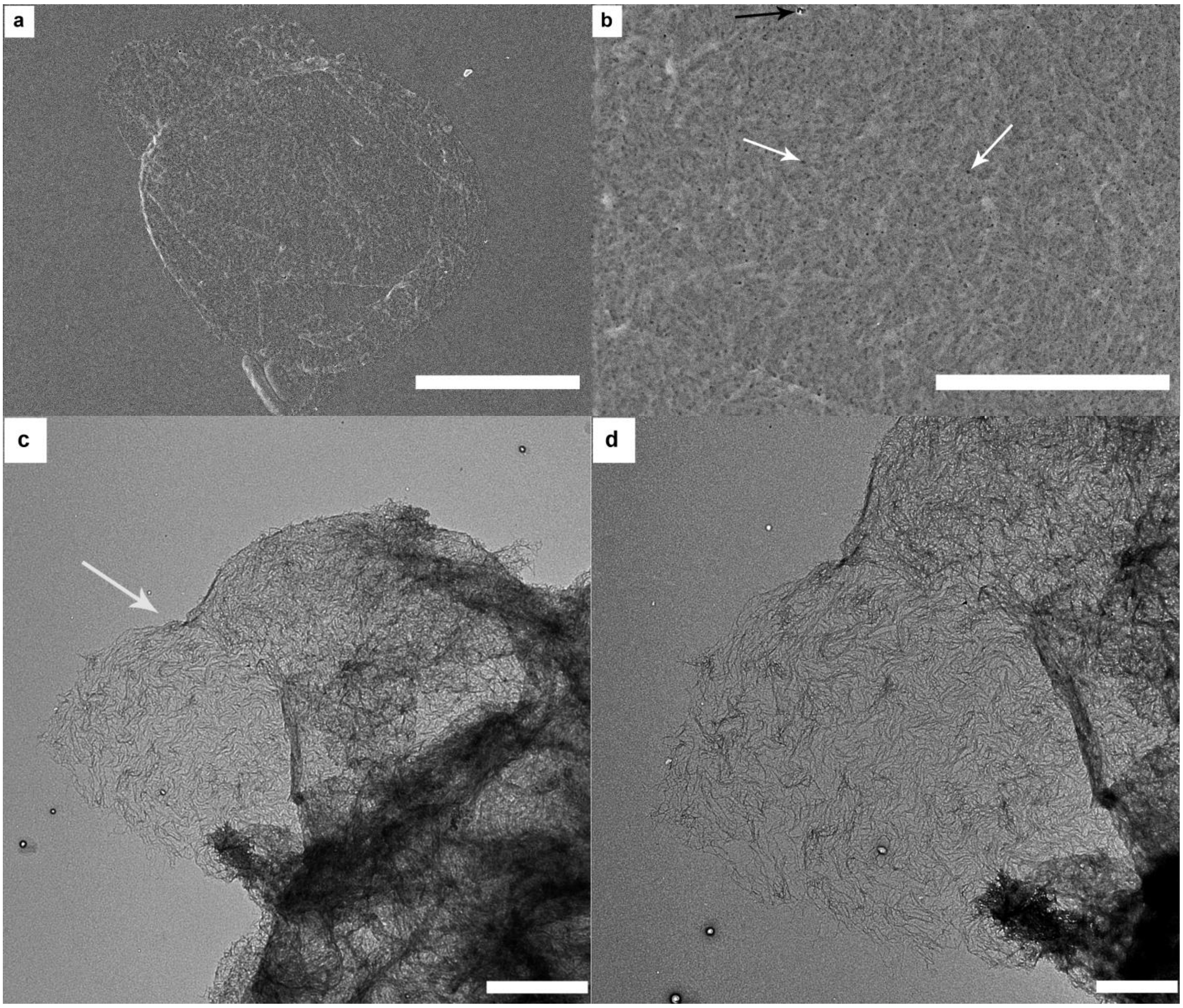

**Supplementary Figure 6. SEM and TEM micrographs of the CNF/pectin wall of expanded plantosomes emptied from their interior lipids.** In (a) and (b), SEM micrographs at two different magnifications. (a) A single collapsed structure of an expanded plantosome devoid of interior lipids. (b) A high-magnification image of the CNF/pectin wall of the structure in (a). The white arrows point to small pores, whereas the black arrow points to a larger pore present in the CNF/pectin wall. In (c) and (d): TEM image of an expanded plantosome that burst during the release of the interior lipids. The arrow points to the burst cavity. In (d), a high-magnification image of the structure in (c), where the CNFs are more clearly visible. TEM micrographs (c-d) are representative of three repeated experiments and images (a-b) are representative SEM micrographs for one of the TEM grids. Scale bars: 5 µm (a, c) and 2 µm (b, d).

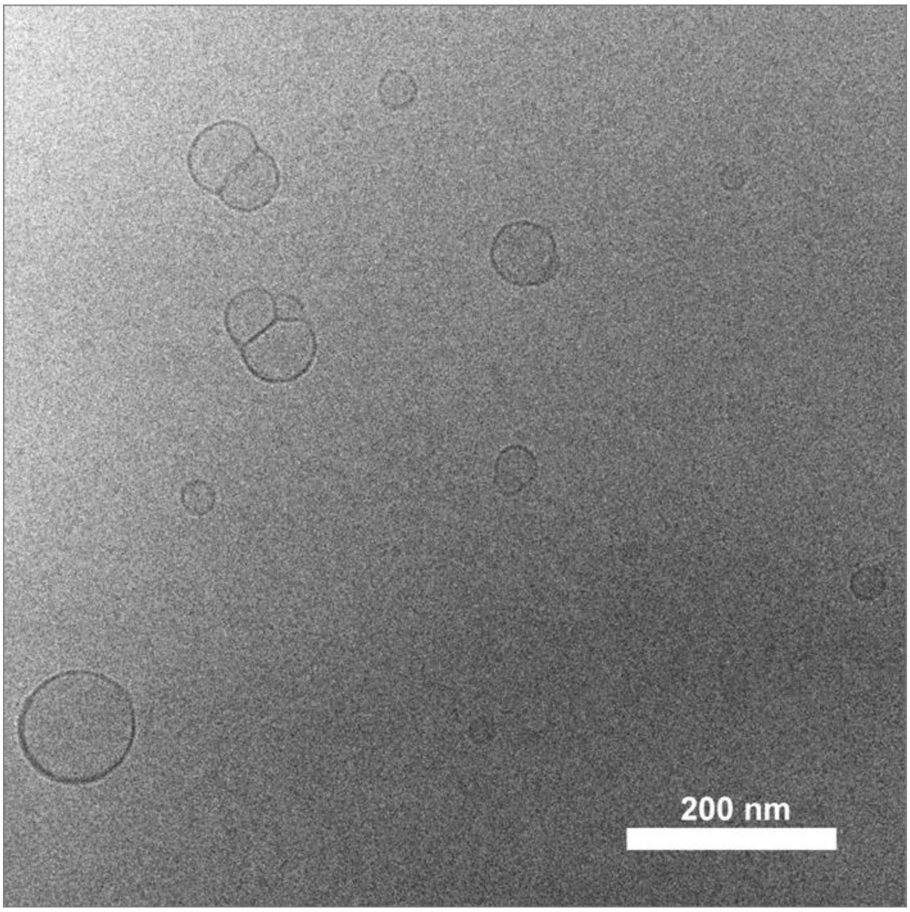


**Supplementary Figure 7**. **OA/oleate/POPC/POPE mixture forms vesicles in buffer.** Cryo-TEM micrograph showing vesicles formed from a lipid mixture containing OA/oleate/POPC/POPE/Rh-DOPE in 0.2 M ammonium acetate and 100 mM NaCl in MilliQ-water. The composition of the lipid mixture was 99.78 mol% OA and 0.18 mol% POPC and 0.035 mol% POPE. The pH was 8.6 (adjusted with ammonia). The lipid preparation was filtered through a 200 nm filter. Representative image of two experiments.

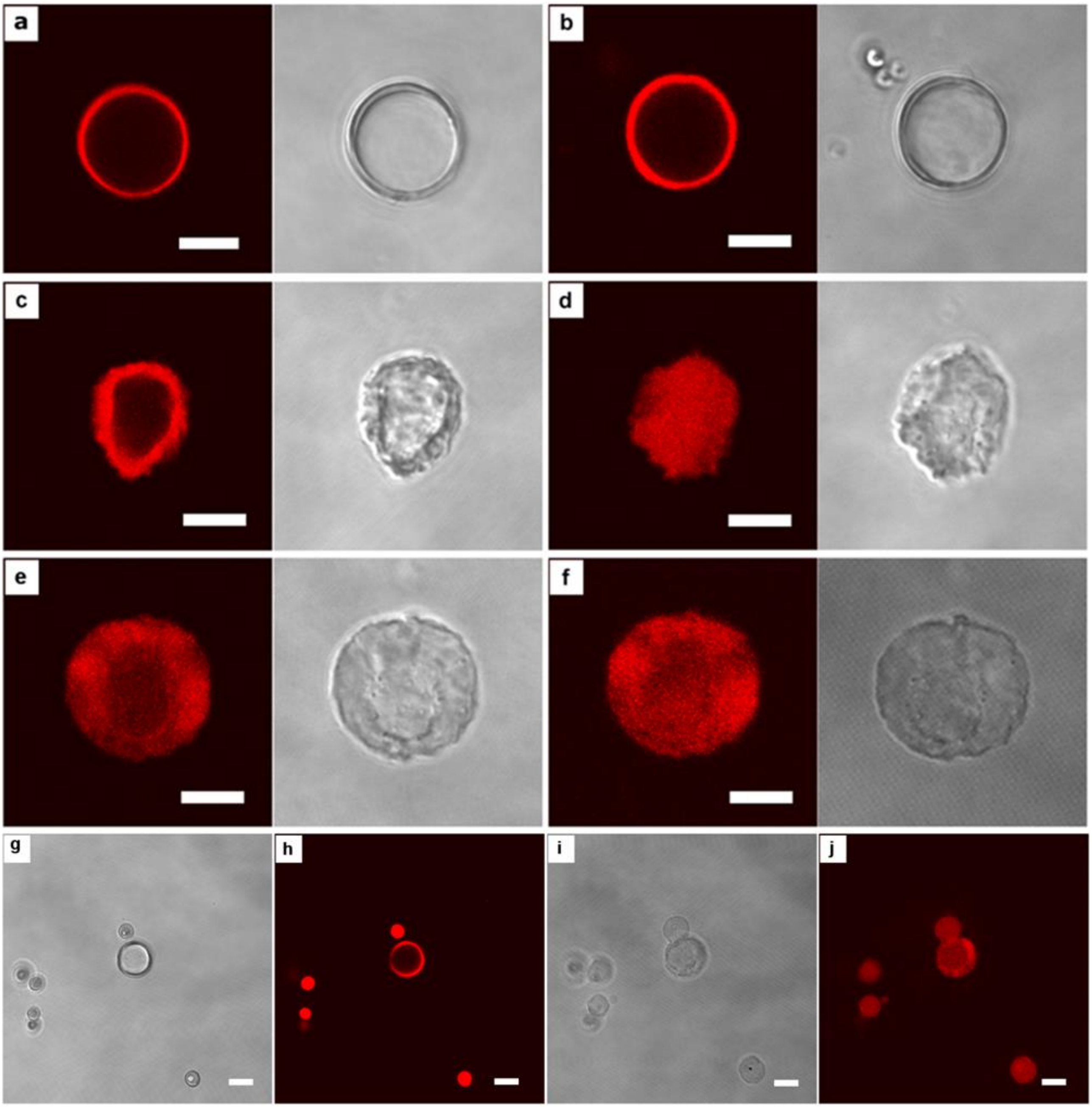


**Supplementary Figure 8**. **Preparation of expanded plantosomes.** CLSM images of a plantosomes at different pH values and media: (a) 100 mM NaCl solution, (b) transferred into 0.19 M ammonium acetate solution (with 100 mM NaCl) at pH 6.5. pH increase to (c) 8.0 (d), 8.3, and finally to (e) 8.6. In (f) after one hour at pH 8.6. (g-j): microcapsules and one plantosome at different pH values: The pH is 6.5 (g and h) and 8.6 (i and j). Both fluorescence and corresponding transmission images are included. The lipid phase contained OA/oleate, POPE, POPC and Rh-DOPE (red). The images fluorescent images are the same as those presented in Fig. 5 of the main manuscript. Scale bars: 10 µm.

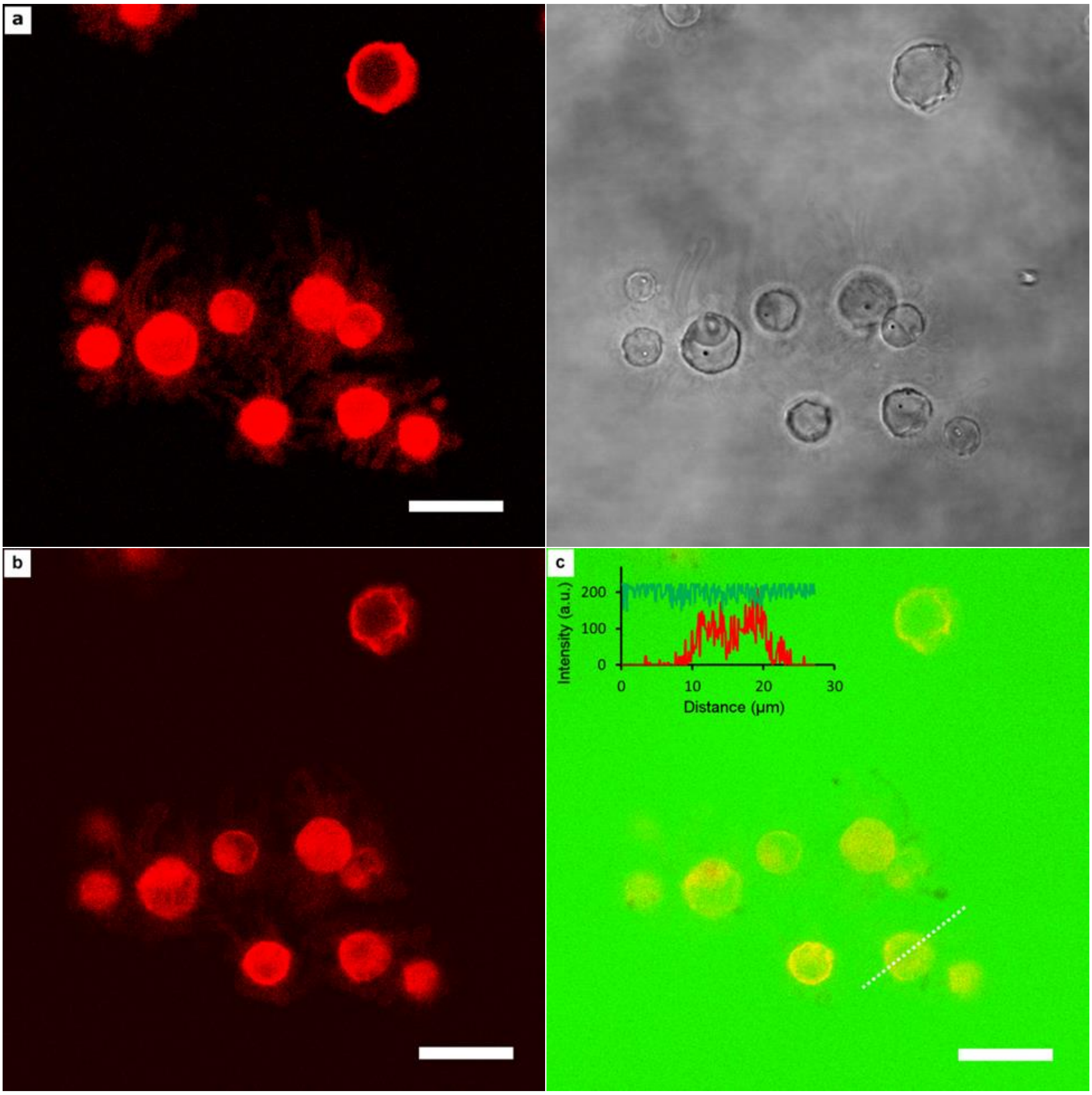


**Supplementary Figure 9**. **Permeability properties of expanded plantosomes**. CLSM images of expanded plantosome structures at pH 8.6 (100 mM NaCl and 0.2 M ammonium acetate) prior to FITC-dextran addition (a) and after the addition of FITC-dextran (b and c). The expanded plantosomes were prepared as described previously and had a capsule wall of CNF/pectin and a core and tubular protrusions containing OA/oleate, POPE and POPC and Rh-DOPE (red). In (b) Rh red channel and (c) combined Rh and FITC channels are shown. The FITC-dextran ($\overline{M}_w$= 4 kDa, 1 mg $mL^{-1}$, green) permeated through the expanded plantosome walls within seconds. The contrast/brightness has been adjusted in (a) and (b) to reveal the tubular protrusions. Inset shows the fluorescence intensity profile of the expanded plantosome dotted in (c). Representative images of three measurements. Scale bars: 20 μm.

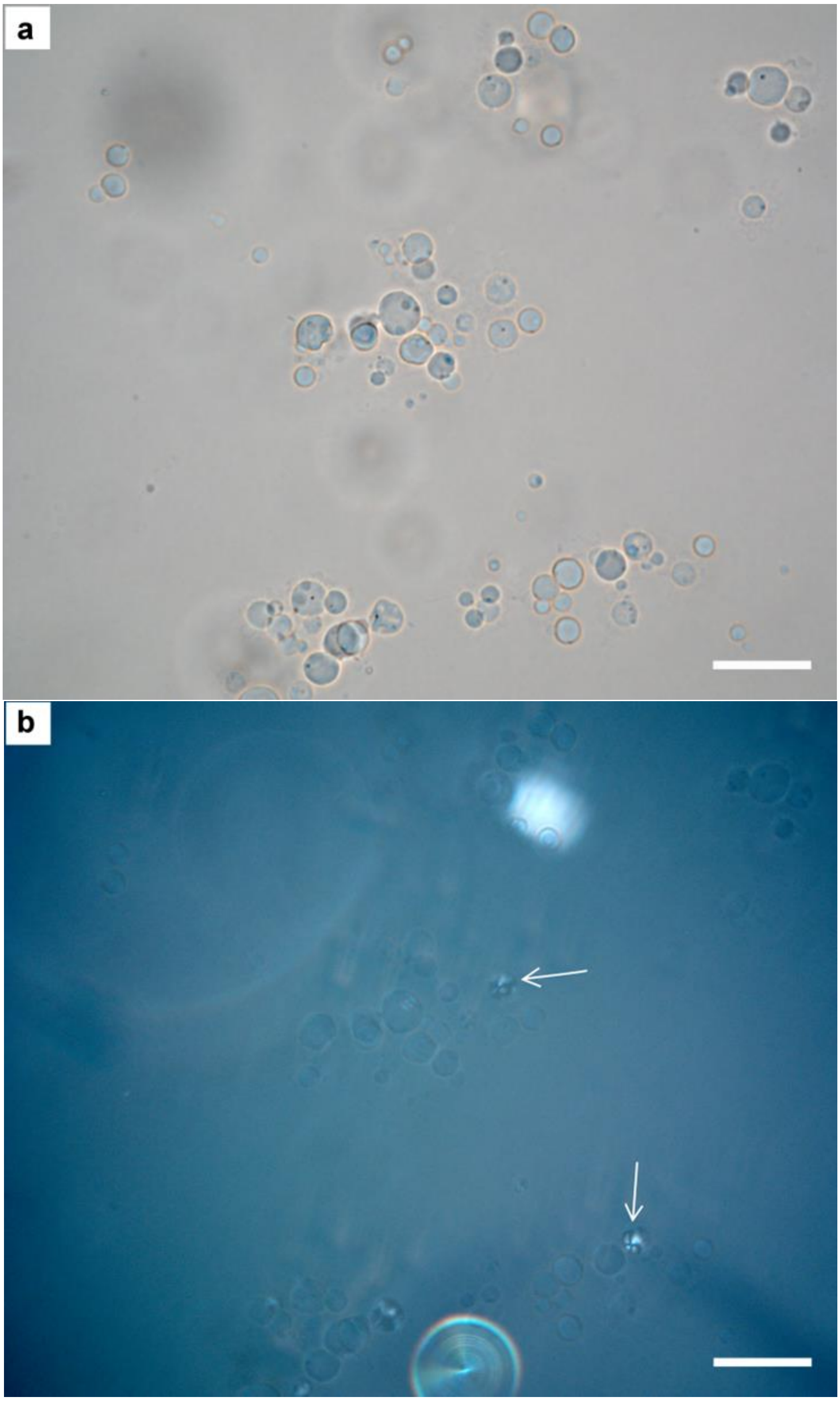


**Supplementary Figure 10. POM of expanded plantosomes.** POM image of expanded plantosomes (pH 8.6, 100 mM NaCl and 0.2 M ammonium acetate) prepared as described previously. These had a CNF/pectin wall and interior lipids (OA/oleate, POPE, POPC and Rh-DOPE). (a) Light microscopy image and (b) the corresponding POM. The arrows point to the very few Maltese crosses observed. Image are representative of five experiments. Scale bars: 50 µm.

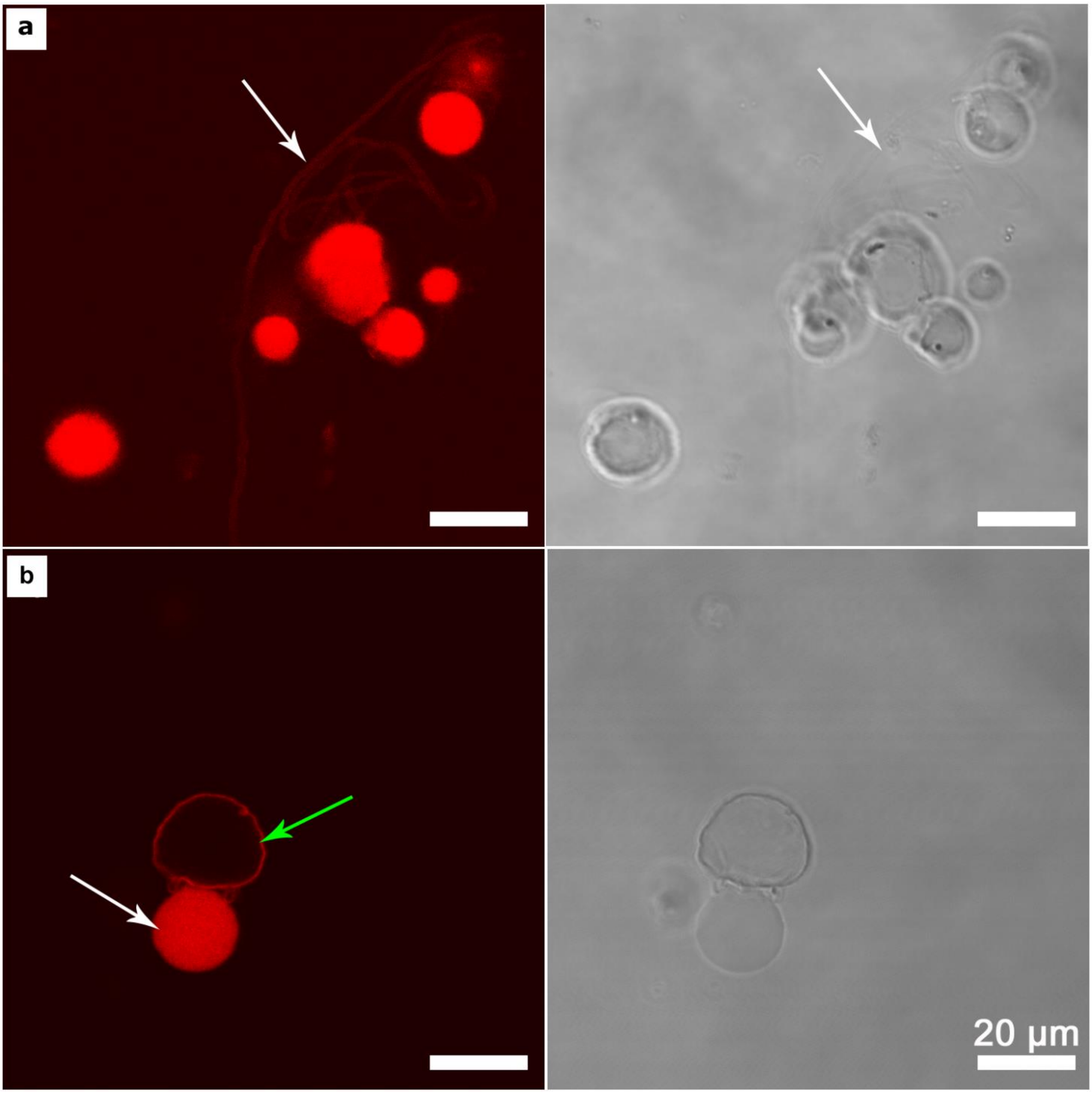


**Supplementary Figure 11. Expanded plantosomes.** CLSM (fluorescence and transmission) images showing (a) lipid tubular protrusions from the surface of expanded plantosomes (pH 8.6, 100 mM NaCl and 0.2 M ammonium acetate, prepared as described previously). These had a CNF/pectin wall and interior lipids (OA/oleate, POPE, POPC and Rh-DOPE (red)). Lipid tubular protrusions were observed in about 80% of the expanded plantosomes (n = 102, obtained from eight experiments). In (b) the capsule wall broke during the expansions, due to the inner pressure, and the lipid content (observed as a large red dot, white arrow) was released. The green arrow points to the CNF/pectin capsule wall which also contained adsorbed lipids. Image (b) is representative of four experiments. Scale bars: 20 μm.

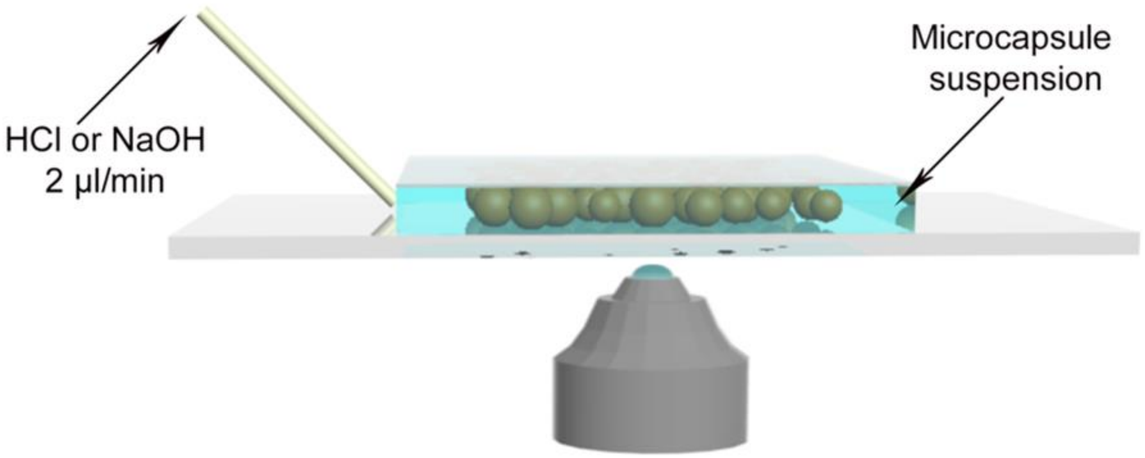


**Supplementary Figure 12. Experimental setup.** Experimental setup to monitor the change in the microcapsules' morphology as well as the lipid organization within microcapsules' OA/oleate cores under acidic and alkaline conditions.

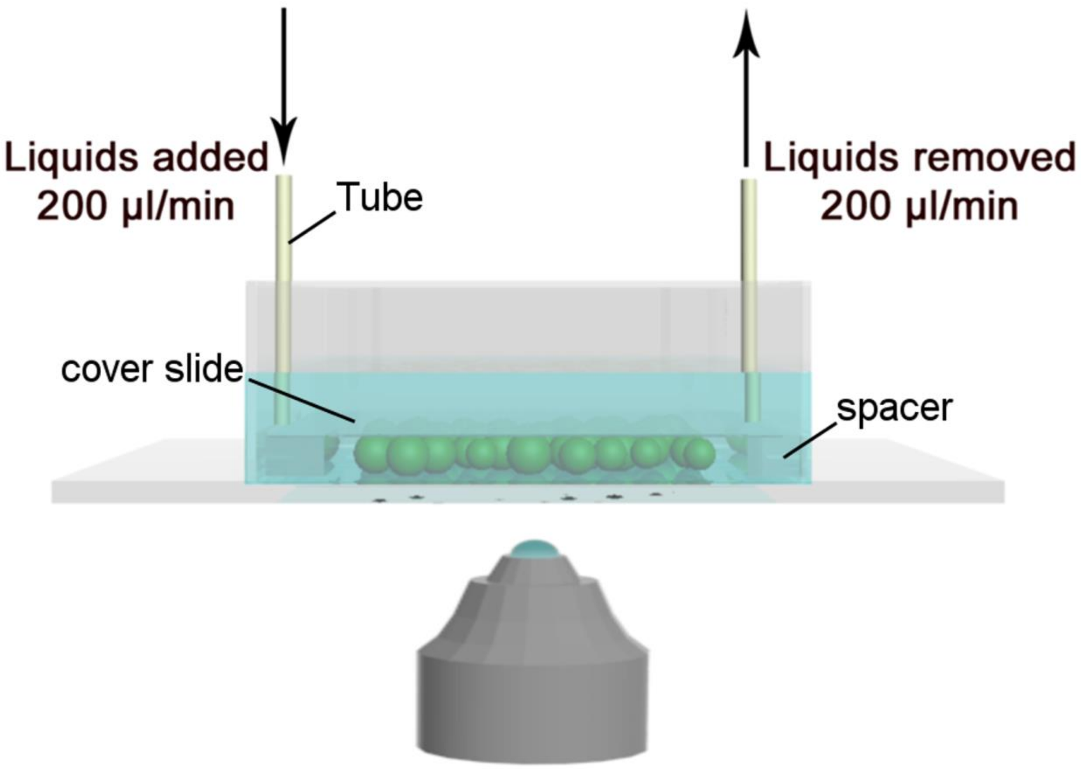


**Supplementary Figure 13. Experimental setup for the modified µ-slide well**. Experimental setup for imaging the plantosomes at different pH, 4 kDa FITC-dextran and different $MgCl_2$ concentration. For details see Methods section.

## Supplementary References


1 Mele, S. *et al.* Phase behavior in the biologically important oleic acid/sodium oleate/water system. *Chemistry and Physics of Lipids* **211**, 30-36 (2018).

2 Suga, K., Kondo, D., Otsuka, Y., Okamoto, Y. & Umakoshi, H. Characterization of aqueous oleic acid/oleate dispersions by fluorescent probes and raman spectroscopy. *Langmuir* **32**, 7606-7612 (2016).

3 Svagan, A. J. *et al.* Liquid-core nanocellulose-shell capsules with tunable oxygen permeability. *Carbohyd Polym* **136**, 292-299 (2016).

4 Parker, R. M. *et al.* Hierarchical self-assembly of cellulose nanocrystals in a confined geometry. *Acs Nano* **10**, 8443-8449 (2016).

5 Edwards, K., Silvander, M. & Karlsson, G. Aggregate structure in dilute aqueous dispersions of oleic acid/sodium oleate and oleic acid/sodium oleate/egg phosphatidylcholine. *Langmuir* **11**, 2429-2434 (1995).

6 Zhu, T. F. & Szostak, J. W. Coupled growth and division of model protocell membranes. *Journal of the American Chemical Society* **131**, 5705-5713 (2009).
