# Supplementary material for: Primary cell wall inspired micro containers as a step towards a synthetic plant cell
Source: Nat Commun. 2020 Feb 19;11:958. doi: 10.1038/s41467-020-14718-x (PMC7031234; doi:10.1038/s41467-020-14718-x)
Supplement: Supplementary file 2 — Reporting summary [file 41467_2020_14718_MOESM2_ESM.pdf]

nature research

Corresponding author(s): Anna J. Svagan

Last updated by author(s): Jan 23, 2020

Reporting Summary

Nature Research wishes to improve the reproducibility of the work that we publish. This form provides structure for consistency and transparency in reporting. For further information on Nature Research policies, see Authors & Referees and the Editorial Policy Checklist .

Statistics

For all statistical analyses, confirm that the following items are present in the figure legend, table legend, main text, or Methods section.

n/a	Confirmed	
[ ]	[x]	The exact sample size (*n*) for each experimental group/condition, given as a discrete number and unit of measurement
[ ]	[x]	A statement on whether measurements were taken from distinct samples or whether the same sample was measured repeatedly
[x]	[ ]	The statistical test(s) used AND whether they are one- or two-sided<br>*Only common tests should be described solely by name; describe more complex techniques in the Methods section.*
[x]	[ ]	A description of all covariates tested
[x]	[ ]	A description of any assumptions or corrections, such as tests of normality and adjustment for multiple comparisons
[ ]	[x]	A full description of the statistical parameters including central tendency (e.g. means) or other basic estimates (e.g. regression coefficient) AND variation (e.g. standard deviation) or associated estimates of uncertainty (e.g. confidence intervals)
[x]	[ ]	For null hypothesis testing, the test statistic (e.g. *F*, *t*, *r*) with confidence intervals, effect sizes, degrees of freedom and *P* value noted<br>*Give P values as exact values whenever suitable.*
[x]	[ ]	For Bayesian analysis, information on the choice of priors and Markov chain Monte Carlo settings
[x]	[ ]	For hierarchical and complex designs, identification of the appropriate level for tests and full reporting of outcomes
[x]	[ ]	Estimates of effect sizes (e.g. Cohen's *d*, Pearson's *r*), indicating how they were calculated

Our web collection on statistics for biologists contains articles on many of the points above.

Software and code

Policy information about availability of computer code

Data collection	Zeiss Zen 2.6 (blue edition), Zeiss Zen Black 2012, LSM 510 3.2 SP2, IS VisiCam Image Analyser 3.9.0.605, NanoScope 8.15, Hitachi HT7700 02.05, S-4800 04.05
Data analysis	Zeiss Zen 2.6 (blue edition) and Zeiss LSM Image Browser version 4.2.0.121, Image J 1.50b, NanoScope 8.15, Gwyddion 2.47, Origin 9.1 and Microsoft Excel 2010.

For manuscripts utilizing custom algorithms or software that are central to the research but not yet described in published literature, software must be made available to editors/reviewers. We strongly encourage code deposition in a community repository (e.g. GitHub). See the Nature Research guidelines for submitting code & software for further information.

Data

Policy information about availability of data

All manuscripts must include a data availability statement. This statement should provide the following information, where applicable:

- Accession codes, unique identifiers, or web links for publicly available datasets
- A list of figures that have associated raw data
- A description of any restrictions on data availability

Data supporting the main findings of this work are available within the paper and its Supplementary Information files. Additional data generated and analyzed during the current study are available from the corresponding author on reasonable request.

Field-specific reporting

Please select the one below that is the best fit for your research. If you are not sure, read the appropriate sections before making your selection.

Life sciences study design

All studies must disclose on these points even when the disclosure is negative.

Sample size	The sample size (or number of experiments) were a minimum of three. Where a calculated mean value (X) is reported (e.g. Fig. 1d, Fig. 3, pore size analysis, results in Fig. 5k and Suppl. Fig 3.), we conclude that the size (n) is large enough to ensure that the probability, P, (Gaussian distribution according to the central limit theorem) is 90 % or higher that the calculated mean value, X, lies within a narrow range (±d) from the population mean, μ, i.e. P(-d ≤ (X-μ) ≤ d) ≥ 90%. With d = 2 μm (for average value reported in Fig. 1d), d = 1 μm (Fig. 3 and Suppl Fig. 3), d = 2 nm (pore size calculation) and d = 5.2% (Fig. 5k).
Data exclusions	No data were excluded.
Replication	New batches of microcapsules and plantosomes were synthesized several times (on more than 10 different occasions) during the course of the present study to ensure reproducibility. The different steps to achieve reproducibility (and also processing parameters that affect reproducibility) during batch production were carefully documented and are included in the experimental section. Attempts at replication of the experimental data were successful.
Randomization	Randomization was not applied to different batches, due to little batch-to-batch variation (our materials are reproducible). Randomization was, however, applied during imaging, by selecting random microcapsules and/or plantosomes.
Blinding	Investigators were not blinded during the course of experiments.

Reporting for specific materials, systems and methods

We require information from authors about some types of materials, experimental systems and methods used in many studies. Here, indicate whether each material, system or method listed is relevant to your study. If you are not sure if a list item applies to your research, read the appropriate section before selecting a response.

Materials & experimental systems

n/a	Involved in the study
☒	☐ Antibodies
☒	☐ Eukaryotic cell lines
☒	☐ Palaeontology
☒	☐ Animals and other organisms
☒	☐ Human research participants
☒	☐ Clinical data

Methods

n/a	Involved in the study
☒	☐ ChIP-seq
☒	☐ Flow cytometry
☒	☐ MRI-based neuroimaging
