# Supplementary material for: Primary cell wall inspired micro containers as a step towards a synthetic plant cell
Source: Nat Commun. 2020 Feb 19;11:958. doi: 10.1038/s41467-020-14718-x (PMC7031234; doi:10.1038/s41467-020-14718-x)
Supplement: Supplementary file 3 — Description of Additional Supplementary Files [file 41467_2020_14718_MOESM3_ESM.docx]

**Description of Additional Supplementary Files**

**File Name:** Supplementary Movie 1

**Description:** Microcapsules with CNF/pectin walls and Oleic acid cores at different pHs.

**File Name:** Supplementary Movie 2

**Description:** Plantosome in ammonium acetate at different pHs.

**File Name:** Supplementary Movie 3

**Description:** Tubular structures observed on the surface of expanded plantosomes.

**File Name:** Supplementary Movie 4

**Description:** Effect of Mg2+ ions on expanded plantosomes.
